# Shear Stress‐Responsive Polymersome Nanoreactors Inspired by the Marine Bioluminescence of Dinoflagellates

**DOI:** 10.1002/anie.202010099

**Published:** 2020-11-12

**Authors:** Omar Rifaie‐Graham, Nikolas F. B. Galensowske, Charlie Dean, Jonas Pollard, Sandor Balog, Micael G. Gouveia, Mohamed Chami, Antoine Vian, Esther Amstad, Marco Lattuada, Nico Bruns

**Affiliations:** ^1^ Adolphe Merkle Institute University of Fribourg Chemin des Verdiers 4 1700 Fribourg Switzerland; ^2^ Department of Pure and Applied Chemistry University of Strathclyde Thomas Graham Building, 295 Cathedral Street Glasgow G1 1XL UK; ^3^ BioEM lab Center of Cellular Imaging and NanoAnalytics (C-CINA) Biozentrum University of Basel Mattenstrasse 26 4058 Basel Switzerland; ^4^ Soft Materials Laboratory Institute of Materials École Polytechnique Fédérale de Lausanne, EPFL-STI-IMX-SMAL MXC 231 Station 12 1015 Lausanne Switzerland; ^5^ Department of Chemistry University of Fribourg Chemin du Musée 9 1700 Fribourg Switzerland; ^6^ Current address: Department of Materials and Department of Bioengineering Institute of Biomedical Engineering Imperial College London Exhibition Road London SW7 2AZ UK

**Keywords:** biocatalysis, bioinspired materials, block copolymers, mechanoresponsive materials, vesicles

## Abstract

Some marine plankton called dinoflagellates emit light in response to the movement of surrounding water, resulting in a phenomenon called milky seas or sea sparkle. The underlying concept, a shear‐stress induced permeabilisation of biocatalytic reaction compartments, is transferred to polymer‐based nanoreactors. Amphiphilic block copolymers that carry nucleobases in their hydrophobic block are self‐assembled into polymersomes. The membrane of the vesicles can be transiently switched between an impermeable and a semipermeable state by shear forces occurring in flow or during turbulent mixing of polymersome dispersions. Nucleobase pairs in the hydrophobic leaflet separate when mechanical force is applied, exposing their hydrogen bonding motifs and therefore making the membrane less hydrophobic and more permeable for water soluble compounds. This polarity switch is used to release payload of the polymersomes on demand, and to activate biocatalytic reactions in the interior of the polymersomes.

## Graphical abstract

Polymersomes with nucleobase pairs in their membrane become transiently permeable for substrates when shear forces are applied. These force‐responsive polymersomes allow to switch on enzymatic reactions by turbulent mixing of vesicle dispersions, which is demonstrated with colorimetric and chemoluminescent reactions, as well as the curing of hydrogels.

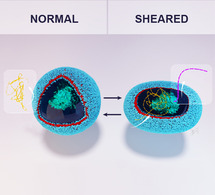

## Introduction

Many biological processes are intimately related to mechanical stimulation.^[1]^ For instance, the sense of touch, hearing, proprioception, and the control of blood pressure are regulated by mechanically responsive proteins that control the flux of ions and other molecules across cell membranes.^[2]^ Unicellular microorganisms are also able to sense stress. For example, some microorganisms perceive their environment through mechanosensing of hydromechanical signals.^[3]^ A captivating example is the marine bioluminescence of dinoflagellates such as *Pyrocystis sp*. or *Noctiluca scintillans*; marine plankton that biochemically produce light when stimulated by hydrodynamic turbulences that are generated by predators, waves, boats, or swimming animals.^[4]^ Mechanosensing events generate an action potential that travels along the vacuole membrane and activates proton channels in the membrane of scintillons, which are specialised organelles for bioluminescence.^[5]^ The influx of protons activates biocatalytic luminescence reactions in the scintillons. On a conceptual level, mechanotransduction leads to a change in permeability of the scintillon wall and therefore an activation of the bioluminescence reaction in the microscale reaction compartments.

Bio‐inspired artificial systems that take this concept as a blueprint are nanoreactors that can be activated by changing the permeability of the reactor shell through mechanical forces. This would allow to switch on chemical reactions on demand by shear stress that is generated, e.g., in flow conditions or during turbulent stirring, and therefore allow to pursue new concepts in catalytic systems engineering. Moreover, shear stress has emerged as a powerful trigger to activate the release of active compounds.^[6]^ For example, mechanical forces can be used to trigger the release of pharmaceutical compounds from drug delivery vehicles,^[7]^ such as vasodilator agents from liposomes at the site of blood vessel stenosis.^[8]^ Concepts to render liposomes permeable by shear are the formation of non‐spherical liposomes,^[8]^ reconstitution of force responsive porins in the membrane,^[9]^ or use of mixed lipid‐surfactant vesicles.^[10]^

Amphiphilic block copolymers are ideally suited to create bio‐inspired nanoreactors because they can be easily functionalised with stimuli‐responsive moieties^[11]^ and because they can self‐assemble into very stable vesicles.^[12]^ These polymersomes have been widely explored as nanocontainers and as nanoreactors that respond to a variety of stimuli,[^12b^, ^13^] such as light,^[14]^ pH,^[15]^ or temperature.^[16]^ Shear stress has been applied to polymersomes to observe mechanical stability and pore formation.[^12a^, ^17^] Polymersomes that can become transiently permeable by shear would be very useful for a variety of applications including drug delivery, selective release of fragrances or of curing agents for 3D printing, and as nanoreactors. To generate shear stress‐responsive polymersomes we decided to implement functional groups into the hydrophobic leaflet of the block copolymer membrane that increase their polarity in response to mechanical perturbation, thus increasing the permeability of the membrane for water‐soluble substrates. We took inspiration from the mechanically induced melting of DNA to achieve this property.^[18]^ Double stranded DNA is hydrophobic inside of its helix because nucleobases form base pairs by hydrogen bonding. In contrast, the nucleobases in single stranded DNA are highly hydrophilic because the unpaired nucleobases form hydrogen bonds to water.^[19]^ Thus, cleavage of nucleobase pairs represents a switch in polarity. Even though DNA motifs have been used as force gauge,[^18e^, ^18f^, ^20^] and polymers that contain nucleobases have been investigated because of their interesting self‐assembly properties,^[21]^ the force‐switchable polarity of nucleobase pairs has not been exploited to tune the permeability of vesicles.

We functionalised amphiphilic block copolymers with a small percentage of nucleobases in their hydrophobic block producing complementary polymers that were self‐assembled into polymersomes. The membrane of these vesicles should be impermeable to water soluble compounds when the nucleobases are paired and permeable when the base pairs are cleaved by mechanical stimulation. Here, we demonstrate that this transient and reversible switch in permeability can be used to control the uptake and release of substances into and from the polymersomes. By encapsulating enzymes into the vesicles, biocatalytic nanoreactors were obtained that could be transiently switched on by hydrodynamic shear stress, for example, to start bioluminescence reactions.

## Results and Discussion

**Figure 1:**
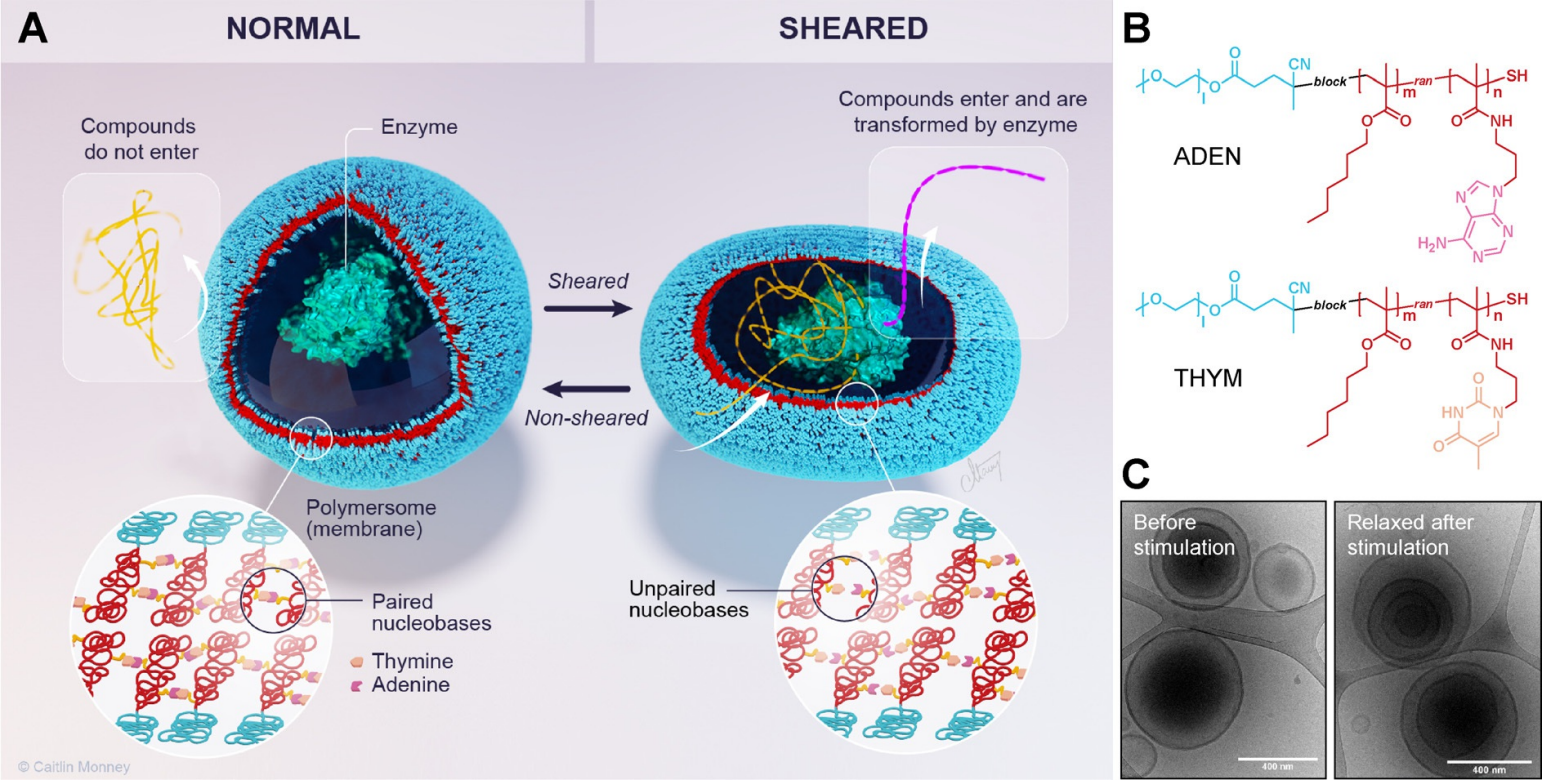
Mechanism of action, and structure of shear‐responsive polymersome nanoreactors. A) Schematic depiction of shear stress‐responsive ADEN/THYM polymersome encapsulating an enzyme. The lower left inset shows the schematic structure of the membrane. It is composed of a bilayer of amphiphilic block copolymers interconnected by a network of nucleobase pairs in the hydrophobic leaflet. When sheared, the polymersome deforms, causing the nucleobase pairs to unpair. The exposed nucleobases increase the polarity of the hydrophobic leaflet and, as a result, the permeability of the membrane to water soluble compounds. They can now cross the membrane and take part in reactions in the polymersome, such as the enzyme‐catalyzed formation of luminescence. (©Caitlin Monney; caitlinmonney.com) B) Chemical structures of the ADEN and THYM polymers. C) Cryo‐TEM images of ADEN/THYM polymersomes before and after shearing. The scale bars correspond to 400 nm.

Block copolymers were synthesized by reversible addition‐fragmentation chain transfer (RAFT) polymerization of 95 mol % hexyl methacrylate and 5 mol % pentafluorophenyl methacrylate from a PEG‐based chain transfer agent. Then, the pentafluorophenyl active esters were used to link either amine modified adenine or thymine to the polymer. Moreover, the same batch of precursor block copolymer was reacted with hexylamine to yield non‐functionalized block copolymers (Synthesis and characterisation of polymers: Supporting Information 1–4). A 1:1 mixture of the two amphiphilic block copolymers that carried either adenine or thymine in their hydrophobic block was self‐assembled into polymersomes, yielding uni‐ and multi‐compartmental vesicles (ADEN/THYM polymersomes) (Characterisation of polymersomes: Figure 1 and Supporting Information 4). Block copolymers that did not contain any nucleobases were also self‐assembled into polymersomes (HEX polymersomes). The self‐assembly of adenine‐functionalized block copolymers or of thymine‐functionalized block copolymers on their own did not result in well‐defined self‐assemblies, possibly because the unpaired nucleobases made the hydrophobic block too polar.

**Figure 2:**
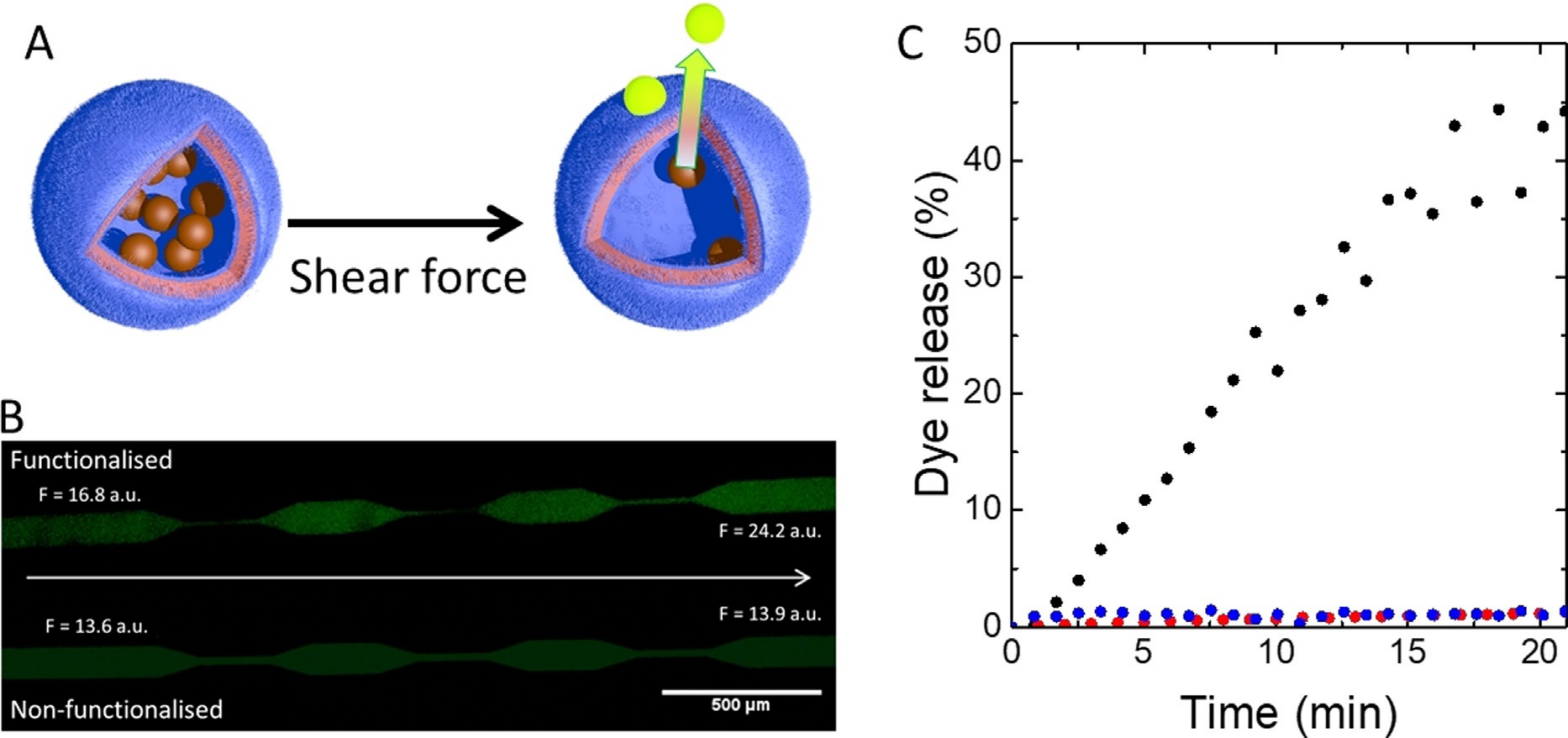
Release of small molecules from shear‐responsive polymersomes. A) Scheme depicting the release of sodium fluorescein from the lumen of the polymersomes if subjected to shear stress. The fluorescent dye was encapsulated at self‐quenching concentrations. Thus, the fluorescence increases when the polymersomes release fluorescein into a dilute environment. B) Fluorescent microscopy images of microfluidic shear flow experiment. A dispersion of sodium fluorescein‐filled ADEN/THYM polymersomes flowed from left to right through a microfluidic device featuring several constrictions (top). Sodium fluorescein‐filled HEX polymersomes were stimulated in the same manner (bottom). The flowrate was of 10 mL h^−1^ (F = fluorescence intensity). Excitation: 495 nm; emission: 519 nm; exposure time: 3 s. C) Release of sodium fluorescein from ADEN/THYM polymersomes (black) and from HEX polymer vesicles (blue) when sheared continuously through a syringe needle at a flowrate of 70 mL min^−1^; release of sodium fluorescein from ADEN/THYM vesicles in the absence of shear flow (red).

The response to shear stress of these polymersomes was assessed by the release of sodium fluorescein from them. We loaded polymersomes containing 5 % of nucleobase residues (ADEN/THYM) in the hydrophobic leaflet with the dye at self‐quenching concentration and introduced them into a microfluidic channel with varying widths (Figure 2 B and Supporting Information 5). An increase of fluorescence was observed as the polymersome dispersion was pushed through multiple constrictions where they were subjected to high shear stresses. This caused the release of the dye, indicating that the vesicle membrane became permeable for sodium fluorescein upon shearing. Polymersomes functionalised with inert hexylamine (HEX) did not yield an increase in fluorescence, indicating that the nucleobases in the hydrophobic leaflet of the polymersome membrane are essential to render the vesicles shear force‐responsive. Release of sodium fluorescein was also monitored using fluorescence spectroscopy (Figure 2 C and Supporting Information 6). In these experiments, polymersomes were continuously sheared by aspirating and ejecting the dispersions through a 0.65 mm diameter syringe needle at a flowrate of 70 mL min^−1^. ADEN/THYM polymersomes released 45 % of their contents within 21 min. The fluorescence did neither increase when ADEN/THYM polymersomes were left unperturbed, nor when HEX polymersomes were sheared. These results support our hypothesis that in the absence of shear stress adenine and thymine form hydrophobic base pairs in the hydrophobic leaflet of the polymersome membrane. When shear is applied, the nucleobases unpair, exposing their polar motifs within the hydrophobic leaflet. This process reduces the hydrophobicity of the hydrophobic leaflet, thereby increasing its permeability.

After ADEN/THYM polymersomes had been mechanically activated, they continued to release fluorescein also in the absence of mechanical forces, albeit at a slower rate than during turbulent mixing (Supporting Information 7). Release accelerated again when the vesicles were sheared again. An explanation for this observation is that the unpairing of nucleobases is reversible, but not complete during the time scale of the experiments.

Shear stress‐induced disassembly of the polymersomes was ruled out by control experiments that demonstrate the integrity of the polymersomes after the shearing process. Cryo‐TEM images suggest that the structure of the polymersomes remained intact after shearing (Figure 1 C). Furthermore, empty nucleobase‐functionalised polymersomes were incubated in a solution of sodium fluorescein. Upon shearing, the polymersomes captured the dye and released it again when sheared (Supporting Information 8).

**Figure 3:**
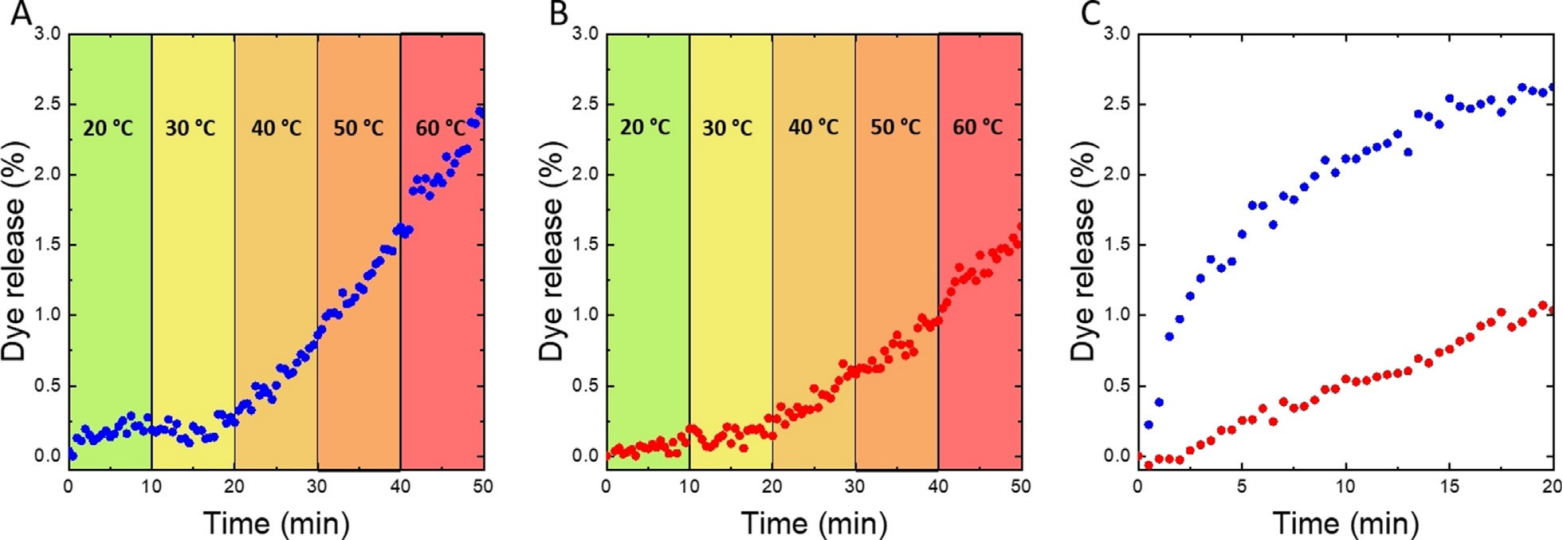
Thermally induced release of sodium fluorescein from ADEN/THYM polymersomes and from HEX polymersomes. A) Temperature ramping induced release of sodium fluorescein from ADEN/THYM polymersomes. B) Temperature ramping induced release of sodium fluorescein from HEX polymersomes. In both panels A and B the temperature was increased step‐wise by 10 °C every 10 min. C) Release of sodium fluorescein at 50 °C from ADEN/THYM polymersomes (blue) and HEX polymersomes (red). In this experiment, dispersions of the dye‐loaded vesicles were directly heated to 50 °C without prior ramping of the temperature.

To prove that the change in permeability of the polymersome membrane was due to the unpairing of nucleobases, we probed the systems with thermal energy to induce nucleobase pair melting. The temperature of polymersome dispersions was ramped from 20 °C to 60 °C in 10 °C steps (Figure 3 A and B). Fluorescein‐filled ADEN/THYM polymersomes were tight at 20 °C and 30 °C, and started to release dye at 40 °C. In contrast, the release of fluorescein from HEX polymersomes increased continuously with temperature. When sodium fluorescein‐filled ADEN/THYM polymersomes were incubated at 50 °C, the initial release of fluorescein was much faster than the initial release of dye from HEX polymersomes at this temperature (Figure 3 C). The results indicate an energy barrier for the thermal melting of the nucleobase pairs. Once nucleobases have unpaired, the polarity of the hydrophobic leaflet of the ADEN/THYM membrane increases. Therefore, the permeability increases. In contrast, release from HEX polymersomes is governed by the thermal increase in diffusion of the cargo through the polymersome membrane.

The threshold shear force for the permeabilisation of the polymersome membrane was determined by fluorescein release studies at various flowrates (Supporting Information 9). ADEN/THYM polymersomes remained impermeable when pushed through a 0.65 mm diameter needle up to a flowrate of 14 mL min^−1^. By contrast, if the flowrate was increased to 15 mL min^−1^, the vesicles became leaky and the dye was released. Taking the geometric configuration of the experimental setup into account, a threshold flowrate of 15 mL min^−1^ leads to Poiseuille flow in the syringe needle, which can be approximated as a simple shear experienced by the polymersomes. Under these conditions, the particles are exposed to compressional and extensional stresses of 10–30 kPa and 20–60 kPa, respectively, for particles with radii in the range of 100–300 nm (Supporting Information 10). Given the quasi‐spherical nature of the polymersomes, and the low stiffness of the hydrophobic leaflet that is mostly composed of poly(hexyl methacrylate),^[22]^ we hypothesise that the differential forces applied along the axes of the polymersomes must induce the elongation of the vesicles, which may unpair the base pairs. In experiments with a flowrate of 70 mL min^−1^, the flow is turbulent and the forces that act on the vesicles are comparably high.

**Figure 4:**
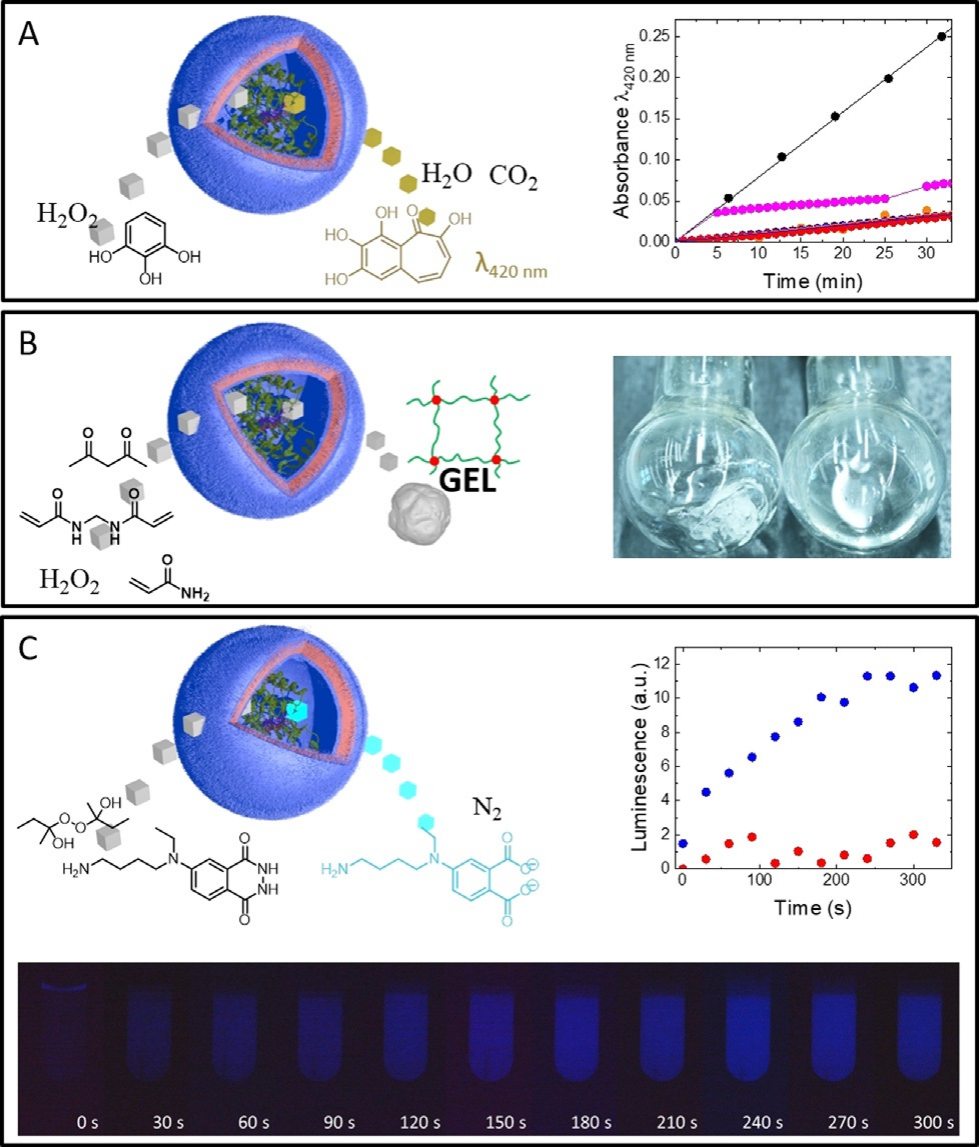
Control of enzymatic reactions by shear‐responsive nanoreactors. A) Left: Pyrogallol assay to measure the activity of HRP‐filled polymersomes. Right: UV/Vis spectroscopic measurement of the formation of purpurogallin catalyzed by continuously sheared HRP‐filled ADEN/THYM polymersomes (•), by HRP‐filled ADEN/THYM polymersomes which were initially sheared for 5 min and then stimulated again after 25 min throughout 5 min (•), by non‐sheared HRP‐filled ADEN/THYM polymersomes (•), by continuously sheared HRP‐filled HEX polymersomes (•), and the blank reaction in the absence of polymersomes and enzymes (•). B) Left: Force‐triggered formation of a polyacrylamide gel by HRP‐filled polymersomes. Right: Photos of a reaction mixture in which a polyacrylamide gel formed within 1 min of ultrasonication in an ice‐cold sonicator bath (left), and of a non‐stimulated reaction mixture containing HRP‐filled ADEN/THYM polymersomes that is still liquid after 24 h at room temperature (right). C) Top left: Luminescence reaction catalyzed by HRP‐filled polymersomes. Top right: Formation of luminescence catalyzed by continuously sheared HRP‐filled ADEN/THYM polymersomes (•), and by continuously sheared HRP‐filled HEX polymersomes (•). Bottom: Photo sequence of a test tube containing the luminescent reaction mixture and HRP‐filled ADEN/THYM polymersomes. The vesicle dispersion was continuously sheared and photographs were taken every 30 s. The brightness of the photos was increased by 50 % for illustration purposes.

An intriguing application of polymersomes is their use as nanoreactors whose catalytic activity can be controlled by external stimuli. To test the suitability of our mechanoresponsive polymersomes to act as nanoreactors, we encapsulated horseradish peroxidase (HRP) into ADEN/THYM polymersomes during vesicle formation. The polymersomes were mechanically activated by repeated aspiration and release through a syringe needle at a flowrate of 70 mL min^−1^, and the catalytic activity was measured by a colorimetric assay based on the HRP‐catalyzed conversion of pyrogallol to purpurogallin (Figure 4 A).^[23]^ The coloured product formed when shear was applied. The reaction stopped as soon as the shearing was interrupted. Thus, the catalytic activity of the nanoreactors could be switched on by turbulent mixing of the vesicle dispersion, and it switched off autonomously when the stimulus was withdrawn. The reaction could be reinitiated when shear was applied again, albeit with a lower apparent activity, possibly because the block copolymers rearrange in such a way as to pair a larger number of complementary nucleobases. The results once again show that the polymersomes retained their integrity during the shear experiments, as in the contrary case the enzyme would have been released and continued to catalyse the reaction in the absence of shear forces. Control experiments with HRP‐filled HEX polymersomes showed no catalytic activity when stimulated under the same conditions.

HRP is known to initiate free radical polymerisations of vinyl monomers in the presence of hydrogen peroxide.^[24]^ Shear‐responsive nanoreactors could therefore be used to cure monomer mixtures in response to mechanical agitation, for example, for the formation of hydrogels on demand or to cure inkjet inks when they pass through a printing nozzle. To demonstrate the formation of solids from aqueous dispersions, ADEN/THYM nanoreactors were added to an aqueous solution of the monomer acrylamide, the crosslinker bisacrylamide, hydrogen peroxide, and the mediator 2,4‐pentanedione. The solution was purged with argon to favour the polymerisation reaction. To avoid the introduction of oxygen during repeated flow through a syringe needle, the dispersions were mechanically stimulated in an ultrasonic bath. Gelation occurred within a period of 1 min (Figure 4 B and Supporting Information Video). In comparison, an unperturbed monomer solution containing HRP‐loaded ADEN/THYM polymersomes did not gel for at least 24 h. A control reaction with HRP‐filled HEX polymersomes did not produce a gel within the same sonication period. These results suggest that the shear‐responsive nanoreactors can be exploited to cure monomer mixtures on demand.

Dinoflagellates produce bioluminescence in response to turbulences in the surrounding medium. Inspired by this biological phenomenon, ADEN/THYM nanoreactors were used to catalyse reactions that result in luminescence. HRP mediates the reaction of a variety of luminol derivatives with peroxides to produce chemoluminescence (Supporting Information 11).^[25]^ Thus, HRP‐loaded ADEN/THYM nanoreactors were used to catalyse the reaction of *N*‐(4‐aminobutyl)‐*N*‐ethylisoluminol (ABEI) with 2‐butanone peroxide in the presence of the luminescence enhancer *p*‐iodophenol (Figure 4 C). Initial luminescence formation was observed but vanished after gentle manual stirring. However, when the reaction mixture was sheared repeatedly through a syringe needle, the dispersion started to emit light, and the luminescence increased with continued shearing. When the mechanical agitation was interrupted, the luminescence decayed. Importantly, conducting luminescence reactions with a variety of substrates enabled probing the selectivity of the polymersome membranes (Supporting Information 12). As control, the reaction was carried out with HRP‐filled HEX polymersomes. Luminescence was very faint and did not increase over time, suggesting once more that the nucleobases are needed to render the polymersome shear‐responsive. Thus, ADEN/THYM nanoreactors can mimic the response of dinoflagellates to shear forces by emitting light when turbulently mixed. A difference between the two luminescent systems is that the emission of dinoflagellate bioluminescence is an intense flash on the timescale of seconds, while the nanoreactors generate faint, but longer lasting luminescence.

## Conclusion

This work on nucleobase‐functionalised polymersomes not only demonstrates that the polarity switch between paired and unpaired base pairs can be used to change the properties of polymeric nanomaterials, but also presents a versatile nanosystem for force‐responsive release and nanoreactor applications. Dinoflagellates have been used as markers to quantify and map shear in fluids.^[26]^ ADEN/THYM nanoreactors could be used for the same purpose but represent a stable alternative to the perishable biological matter. Furthermore, the shear‐responsive polymersomes have the potential to unlock limitations of three‐dimensional inkjet printing.^[27]^ ADEN/THYM polymersomes could be activated by the flow through nozzles of inkjet printers in order to trigger curing reactions of polymerisable inks and bioinks. As the hydrophilic block of the polymers is poly(ethylene glycol), the polymersomes should be biocompatible and could be implemented as drug delivery systems, or to prepare drugs from prodrugs triggered by shear forces that occur in stenosed arteries, during cell mitosis of rapidly dividing cells, or during sonotherapy. Finally, the advantage of synthetic nanosystems is that they can be easily modified during their synthesis. It is easy to envision that the polymersome membranes could be functionalised with other hydrogen bonding motifs to adjust the threshold shear forces to specific applications, or to increase the response to shear by generating more drastic changes in permeability.

## Conflict of interest

O.R.‐G. and N.B. are named inventors on a patent application on force‐responsive polymersomes. All other authors declare no competing financial interests.

## Supporting information

As a service to our authors and readers, this journal provides supporting information supplied by the authors. Such materials are peer reviewed and may be re‐organized for online delivery, but are not copy‐edited or typeset. Technical support issues arising from supporting information (other than missing files) should be addressed to the authors.

Supplementary

Supplementary
