# Supplementary material for: Shear Stress‐Responsive Polymersome Nanoreactors Inspired by the Marine Bioluminescence of Dinoflagellates
Source: Angew Chem Int Ed Engl. 2020 Nov 12;60(2):904–9. doi: 10.1002/anie.202010099 (PMC7839717; doi:10.1002/anie.202010099)
Supplement: Supplementary file 1 — Supplementary [file ANIE-60-904-s001.pdf]

Angewandte Chemie
Eine Zeitschrift der Gesellschaft Deutscher Chemiker

Supporting Information

Shear Stress-Responsive Polymersome Nanoreactors Inspired by the Marine Bioluminescence of Dinoflagellates

*Omar Rifaie-Graham, Nikolas F. B. Galensowske, Charlie Dean, Jonas Pollard, Sandor Balog, Micael G. Gouveia, Mohamed Chami, Antoine Vian, Esther Amstad, Marco Lattuada, and Nico Bruns**

anie_202010099_sm_miscellaneous_information.pdf
anie_202010099_sm_Video.avi

Author Contributions

O.R. Conceptualization: Lead; Data curation: Equal; Investigation: Lead; Methodology: Equal; Visualization: Lead; Writing—Original Draft: Lead; Writing—Review & Editing: Equal
N.G. Investigation: Supporting; Writing—Review & Editing: Supporting
C.D. Investigation: Supporting; Writing—Review & Editing: Supporting
J.P. Methodology: Supporting; Writing—Review & Editing: Supporting
S.B. Formal analysis: Supporting; Investigation: Supporting; Writing—Review & Editing: Supporting
M.G. Investigation: Supporting; Writing—Review & Editing: Supporting
M.C. Investigation: Supporting; Writing—Review & Editing: Supporting
A.V. Investigation: Supporting; Writing—Review & Editing: Supporting
E.A. Formal analysis: Supporting; Methodology: Supporting; Resources: Supporting; Supervision: Supporting; Writing—Review & Editing: Supporting
M.L. Formal analysis: Lead; Methodology: Supporting; Writing—Review & Editing: Supporting
N.B. Conceptualization: Lead; Data curation: Equal; Funding acquisition: Lead; Methodology: Equal; Project administration: Lead; Resources: Lead; Supervision: Lead; Writing—Original Draft: Equal; Writing—Review & Editing: Equal.

Supporting Information

Table of Contents

Experimental Section 3

Materials and equipment 3

Synthesis of activated ester amphiphilic block copolymers 4

Synthesis of primary amine modified nucleobases 4

Post-polymerisation modification of activated ester block copolymers with compounds 5, 6 and hexylamine to yield ADEN (7), THYM (8), and HEX (9) 5

Self-assembly of empty polymersomes and of sodium fluorescein-filled polymersomes 6

Self-assembly of nanoreactors 6

Preparation of empty polymersomes for cryo-TEM 6

Mechanical stimulation of dispersions containing polymersomes for fluorescence spectroscopy, UV-Vis spectroscopy and luminescence experiments 7

Uptake of sodium fluorescein by ADEN/THYM polymersomes and release 7

Determination of the influence of shear in microfluidics devices 7

Thermal release of sodium fluorescein from ADEN/THYM and HEX polymersomes 7

Colorimetric assays catalysed by HRP-containing nanoreactors 8

Chemoluminescence reactions catalysed by HRP-containing nanoreactors 8

Gel formation catalysed by HRP-containing nanoreactors 9

Supporting Results and Discussion 10

Supporting Information 1. Synthesis and characterization of ADEN/THYM and HEX block copolymers 10

Supporting Information 2. Determination of the degree of functionalisation by ^{19}F-NMR spectroscopy 17

Supporting Information 3. Determination of number average molar mass, block ratio and degree of functionalisation by ^{1}H-NMR spectroscopy 17

Supporting Information 4. Self-assembly and characterisation of self-assembled structures 19

Supporting Information 5. Determination of shear stress in the microfluidic device 20

Supporting Information 6. Sodium fluorescein release studies 21

Supporting Information 7. Intermittent shearing of fluorescein-loaded polymersomes 21

Supporting Information 8. Shear flow-induced uptake of sodium fluorescein into ADEN/THYM polymersomes and subsequent release of the dye upon shearing 22

Supporting Information 9. Determination of threshold activation shear flow of ADEN/THYM polymersomes 22

Supporting Information 10. Estimation of the shear forces acting in the syringe and needle 23

Supporting Information 11. Chemoluminescent reactions of HRP 27

Supporting Information 12. Selectivity of the polymersome membrane towards substrates of HRP 27

Explanation of Supporting Information Video 30

Supporting References 30

Experimental Section

Materials and equipment

2,2′-Azobis(2-methylpropionitrile) (AIBN) was purchased from Sigma Aldrich and was recrystallised from methanol. Methanol (≥ 99.8 %), dimethyl sulfoxide (≥ 99.5 %), *N*,*N*-dimethylformamide (DMF) (anhydrous, 99.8 %), tetrahydrofuran (THF) (≥ 99.9 %), 1,4-dioxane (anhydrous, ≥ 99.5 %), dichloromethane (DCM) (≥ 99.8 %), *tert*-butyl methyl ether (99 %), 2,6-lutidine (98 %), *N*-(3-dimethylaminopropyl)-*N*′-ethylcarbodiimide hydrochloride (EDCI) (crystalline), potassium tert-butoxide solution (1 M in THF), tetrabutylammonium bromide (≥ 99 %), magnesium sulphate (≥ 99.5 %), adenine (≥ 99 %), thymine (≥ 99 %), sodium hydride (dry, 95 %), potassium bisulphate (≥ 99 %), basic aluminium oxide (≥ 98 %), ammonium sulphate (≥ 99 %), potassium carbonate (≥ 99 %), sodium chloride (≥ 99 %), silica gel (high-purity grade, pore size 60 Å, 70-230 mesh), sodium phosphate dibasic (≥ 99 %), sodium phosphate monobasic (99 %), triethylamine (Et_3N) (≥ 99.5 %), potassium carbonate (≥ 99 %), hydrocholoric acid (HCl) (37 %), fluorescein sodium salt (BioReagent), methacryloyl chloride (≥ 97 %), di-*tert*-butyl dicarbonate (99 %), hexyl methacrylate (98 %), pyrogallol (≥ 98 %), Sepharose®2B (60-200 µm bead diameter), peroxidase from horseradish (HRP) (Type VI, essentially salt-free, lyophilized powder, 256 units mg^{-1} solid (Unit definition: One unit will form 1.0 mg purpurogallin from pyrogallol in 20 s at pH 6.0 at 20 °C)), pentafluorophenol (≥ 99 %), 4-cyano-4-(phenyl carbonothioylthio) pentanoic acid (> 97 %), 2,6-di-*tert* butyl-4-methylphenol (≥ 99 %), 2,4-pentanedione (≥ 99 %), potassium phosphate monobasic (≥ 99 %), sodium phosphate dibasic (≥ 99 %), poly(ethylene glycol) methyl ether (average M_n≈2000 g mol^{-1}, determined by provider), luminol (97 %), *N*-(4-Aminobutyl)-*N*-ethylisoluminol (≥ 90 %), isoluminol (98 %), and 2-butanone peroxide (technical grade) were purchased from Sigma Aldrich and were used as received. Hexylamine (99 %), and 4-dimethylaminopyridine (DMAP) (99 %) were purchased from Acros Organics and were used as received. Hydrogen peroxide (35 %) was purchased from Reactolab SA and was used as received. Deuterated solvents (D_2O, $CDCl_3$, DMSO-d_6) were purchased from Cambridge Isotope Laboratories, Inc. 30 % acrylamide/Bis acrylamide solution, 37.5:1 was purchased from BioRad. Ultrapure water was obtained from Purelab Flex II (Veolia water system) at 18.2 mΩ using an LC208 purification pack.

An Analytik Jena Specord 50 Plus spectrophotometer was used to produce UV-Vis measurements.

Unless otherwise stated, fluorescence spectroscopy was performed with a Varian Cary Eclipse Fluorescence Spectrometer equipped with a Varian Cary Single Cell Peltier Accessory at 20 °C. Fluorescence emission spectra of sodium fluorescein were recorded with an irradiation wavelength of 494 nm (excitation slit width: 5 nm; emission slit width: 5 nm), with an emission wavelength range of 450 nm to 650 nm, and a scan rate of 10 nm s^{-1}.

NMR spectroscopy was carried out at 297.2 K on a Bruker Avance DPX 400 spectrometer at frequencies 400.19 MHz for ^{1}H nuclei, 376.51 MHz for ^{19}F nuclei and 100.63 MHz for ^{13}C nuclei. The spectra were referenced internally with residual solvent peaks.

GPC samples were run using THF at 30 °C as the mobile phase at a flowrate of 1 ml min^{-1}. The stationary phase consisted of an Agilent 1200 series HPLC system equipped with an Agilent PLgel mixed guard column (particle size = 5 µm) and two Agilent PLgel mixed-D columns (ID = 7.5 mm, L = 300 mm, particle size = 5 µm). The readout signals were recorded by an Optilab REX interferometric refractometer (Wyatt Technology Corp.). The resulting raw data was analysed on Astra (Wyatt Technology Corp.) and molecular weights were determined based on narrow-molecular-weight polystyrene calibration standards (from 2340 to 275300 g mol^{-1}).

TEM experiments were carried out on a FEI Tecnai Spirit operating at a voltage of 120 kV and equipped with a side-mounted Veleta camera. The sample was dried overnight at room temperature. 5 µL of a 2 % ammonium molybdate solution were blotted on the carbon-coated copper grid and were removed with a filter paper after 15 s. Particle size analysis was performed using ImageJ (v1.46r).

For cryo-TEM imaging, a 4 µL aliquot of the sample was adsorbed onto a holey carbon-coated grid (Lacey, Tedpella, USA), blotted with Whatman 1 filter paper and vitrified into liquid ethane at -180 °C using a Leica GP plunger (LEICA Microsystems, Austria). Frozen grids were transferred onto a Philips CM200FEG electron microscope (Philips, Netherlands) using a Gatan 626 cryo-holder (GATAN, USA). Electron micrographs were recorded at an accelerating voltage of 200 kV and a nominal magnification of 50000 x with a pixel size of 2.2 Å at the sample level, using a lowdose system (30 e-/Å2) and keeping the sample at -175 °C. Defocus values were -4 to -5 µm. Micrographs were recorded with a 4K x 4K XF416 CMOS camera (TVIPS, Germany).

Dynamic light scattering (DLS) and static light scattering (SLS) data were obtained with 2 mL of a dilute aqueous dispersion of the polymersomes. Data were collected at constant temperature (21 °C) on a commercial goniometer instrument (3D LS Spectrometer, LS Instruments AG, Switzerland) and analysed employing previously reported analyses.[1]

Synthesis of activated ester amphiphilic block copolymers

Polymers consisting of poly(ethylene glycol)-*b*-(poly(hexyl methacrylate)-*co*-poly(pentafluorophenyl methacrylate)) were synthesised by chain extension of poly(ethylene glycol) 4-cyano-4-(phenylcarbonothioylthio)pentanoate (**1**) by Reversible Addition-Fragmentation Chain Transfer (RAFT) radical polymerisation according to a previously described method.[2] To this end, AIBN (1.5 mg, 9.14 µmol) was weighted together with **1** (146.2 mg, 73.1 µmol) and dissolved in 1.15 mL of 1,4-dioxane. Hexyl methacrylate (HMA) and pentafluorophenyl methacrylate (PFPMA) (**2**) were purified from their quinone-based polymerisation inhibitors by means of a basic aluminium oxide plug. 856 µL (0.74 g, 4.34 mmol) and 41.3 µL (57.6 mg, 0.23 mmol) of the monomers were added to the polymerisation solution, respectively. The ratio I:CTA:M was 1:8:500, being the ratio HMA:PFPMA 95:5 (Supporting Information Table 1). The solution was bubbled with argon for 1h. The reaction was initiated by subjecting it to 90 °C and was carried for 2h. The reaction was quenched by exposing it to atmospheric oxygen and the polymer was precipitated in a 60:40 methanol/water solution. The resulting suspension was centrifuged at 5000 g for 30 min at 0 °C (Heraeus Megafuge 16R, TX-400 x 400 mL Swinging Bucket Rotor; Thermo Scientific). The resulting pellets were dried in a vacuum oven at 40 °C overnight.

Synthesis of primary amine modified nucleobases

3-(Boc-amino) propyl bromide (**4**) was synthesised by modifying a protocol from Brouwer et al. (Supporting Information Scheme 1).[3] (3-bromopropyl) amine hydrobromide (11.7 g, 53.4 mmol) and di-*tert*-butyl dicarbonate (11.7 g, 53.6 mmol) were suspended in 54 mL of DCM and cooled down to 0 °C. Then, triethylamine (8.94 mL, 63.6 mmol) was added drop-by-drop over a period of 3 h. After stirring at room temperature for 1 day, the solution was successively washed with 25 mL of 1 M potassium bisulphate solution, 25 mL ultrapure water, and 25 mL of brine. The organic phase was dried *in vacuo* and the resulting oil was dissolved in hexane. The product was purified by passing it through a silica gel plug and the solvent was evaporated to yield a colourless oil which crystallised at 4 °C (79 %, 10.077 g). ^{1}H NMR ($CDCl_3$): δ/ppm 4.61 (1H, b), 3.41 (2H, t), 3.24 (2H, t), 2.01 (q, 2H), 1.41 (s, 9H).

^{13}C NMR ($CDCl_3$): δ/ppm 156.06, 79.50, 39.1, 32.82, 30.89, 28.49. The carbon and proton assignation and the integrals can be found in Supporting Information Fig. 1.

3-(Adenine-9-yl) propylamine (**5**) was synthesised by modifying a procedure previously described by Spijker et al. (Supporting Information Scheme 1).[4] Adenine (7.832 g, 57.96 mmol) and sodium hydride (1.55 g, 64.5 mmol) were introduced in a round bottom flask and subjected to 3 argon-vacuum cycles. 300 mL of anhydrous dimethyl formamide (DMF) were added and the dispersion was stirred for 1 h until no more hydrogen gas was produced. Then, a solution of 3-(Boc-amino) propyl bromide (17.91 g, 75.19 mmol) in 90 mL of anhydrous DMF was added drop-by-drop. After 2 days of reaction at room temperature, the solvent was evaporated *in vacuo* and the resulting solid was dissolved again in a mixture of 700 mL DCM and 300 mL water. The organic fraction was washed 3 times with 300 mL water and was then dried on a rotary evaporator. The resulting oil was dissolved in 250 mL of a 2 M HCl solution in methanol to allow the deprotection of the primary amine. As the reaction proceeded, the desired product precipitated from the solution. The product was centrifuged at 5000 g for 20 min (Thermo Scientific, Heraeus Megafuge 16R, TX-400 x 400 mL Swinging Bucket Rotor). The resulting pellets were resuspended 3 times in methanol and centrifuged in the same manner. The product was dried overnight *in vacuo* at 40 °C. A white solid was obtained (5.76 g, 52 %). ^{1}H NMR (D_2O): δ/ppm 8.44 (s, 1H), 8.37 (s, 1H), 4.44 (t, 2H), 3.06 (t, 2H), 2.29 (q, 2H). ^{13}C NMR (D_2O): δ/ppm 149.73, 148.63, 141.70, 144.34, 118.11, 41.50, 36.68, 27.25. The carbon and proton assignation and the integrals can be found in Supporting Information Fig. 2.

To synthesise 3-(thymine-1-yl) propyl amine (**6**), a procedure also described by Spijker et al.[4] was modified (Supporting Information Scheme 1). Thymine (11.838 g, 93.86 mmol), tetrabutyl ammonium bromide (1.918 g, 5.95 mmol) and potassium carbonate (13.065 g, 94.53 mmol) were jointly subjected to 3 vacuum-argon cycles and were then suspended in 560 ml of anhydrous DMF. 3-(Boc-amino) propyl bromide (15.348 g, 64.45 mmol) was dissolved in 100 mL of anhydrous DMF and was added to the previous dispersion. The reaction was stirred at room temperature for 48 h and was quenched with 82 mL water followed by the evaporation of solvents *in vacuo*. The resulting solid was dissolved in 1 L of a 1:1 DCM/water mixture. The organic phase was washed 3 times with ultrapure water and was then evaporated to dryness. The resulting oil was dissolved in 200 mL of a 2 M HCl solution in methanol and let to react overnight. The desired product precipitated throughout the reaction and was purified in the same way as 3-(adenine-9-yl) propylamine yielding a white solid (3.65 g, 31 %). ^{1}H NMR (DMSO-d_6): δ/ppm 7.61 (s, 1H), 3.73 (t, 2H), 2.77 (t, 2H), 1.89 (q, 2H), 1.75 (s, 3H). ^{13}C NMR (DMSO- d_6): δ/ppm 164.25, 151.04, 141.25, 108.71, 44.58, 36.15, 26.71, 11.94. DEPT-135 (DMSO- d_6): δ/ppm 141.00, 44.32, 35.88, 26.45, 11.68. The carbon and proton assignation and the integrals can be found in Supporting Information Fig. 3.

Post-polymerisation modification of activated ester block copolymers with compounds 5, 6, and hexylamine to yield ADEN (7), THYM (8), and HEX (9)

Every batch of the activated ester block copolymer **3** was divided into 3 aliquots, which were independently reacted with **5**, **6** or *n*-hexylamine to yield ADEN (**7**), THYM (**8**) and HEX (**9**) (Supporting Information 2). For the synthesis of **7** and **8**, 100 mg of **3** were dissolved in 2 mL of DMF. In a separate round bottom flask **5** (115 mg, 0.598 mmol) or **6** (110 mg, 0.600 mmol) were dissolved in 4 mL of dimethyl sulfoxide (DMSO) at 90 °C. Then, 0.592 mL of a 1 mol L^{-1} potassium *tert*-butoxide solution in THF was added. Finally, the polymer solution was added. The reaction was allowed to proceed for 6 days at 90 °C. The conversion from the activated ester to amide formation was followed by ATR-FT-IR

(Supporting Information Fig. 4). If a reaction was not complete, additional 0.2 mL of the 1 mol L^{-1} potassium *tert*-butoxide solution in THF was added and the reaction was ended when completion was achieved. The reactions were ended by pouring them into a saturated solution of $(NH_4)_2SO_4$. The dispersions were centrifuged to form a pellet or a floating solid. The solid was filtered from the solution and redissolved in THF. The resulting solution was filtered again to remove the precipitated salts. Then, the solution was dialysed against 1 L of THF to remove the pentafluorophenol that had formed during the amidation (Spectra/Por 6 standard regenerated cellulose, pre-wetted dialysis tubing molecular weight cut-off = 1000 g mol^{-1}). The solution was filtered and evaporated *in vacuo*. The polymer was dried in a vacuum oven at 40 °C overnight.

In the case of the functionalisation with hexylamine, 100 mg of **3** were dissolved in 4 mL DMF and then triethylamine (1.00 ml, 7.17 mmol) was added. *n*-Hexylamine (1.00 mL, 7.61 mmol) was added and reacted for 3 days at 60 °C. The polymers were purified in the same way as the polymers functionalised with nucleobases.

The GPC and ^{1}H NMR data of the postfunctionalised polymers is summarised in Table 1.

Self-assembly of empty polymersomes and of sodium fluorescein-filled polymersomes

Unless otherwise stated, 0.7 mg of THYM and 0.7 mg of ADEN of the same initial batch were dissolved together in 1.5 mL of THF in a round bottom flask. The solvent was slowly evaporated on a rotary evaporator to yield a thin film. Then, 1.5 mL of ultrapure water was added and the round bottom flask was placed on an ultrasound bath overnight. For dye release studies, the polymersomes were self-assembled in 1.5 mL water that contained 14.1 mg (37.5 µmol) sodium fluorescein, *i.e.* at a self-quenching concentration of the dye. As it was envisioned that the size of the polymersomes could affect the response to shear, the systems were homogenised for this set of experiments. Therefore, ADEN/THYM polymersomes were successively extruded through 0.4 µm and 0.1 µm track-etched polycarbonate membranes (Whatman) and were then dialysed (Spectra/Por 6 standard regenerated cellulose, pre-wetted dialysis tubing molecular weight cut-off = 1000 g mol^{-1}) against 1 L of ultrapure water for 3 days. The water was exchanged each day once. The polymersomes were further purified from remaining free dye and micelles by size exclusion chromatography on Sepharose 2B using ultrapure water as the mobile phase.

Self-assembly of nanoreactors

2.5 mg of THYM and 2.5 mg of ADEN were dissolved together in 2 mL of THF in a round bottom flask. The solvent was slowly evaporated on a rotary evaporator to generate a thin film. Then, 2 mL of a solution of HRP ($7.95 \cdot 10^{-5}$ M) in 0.1 M phosphate buffer pH 6.6 were introduced into the round bottom flask, which was placed in an ice-cold ultrasound bath for a period of 6 h. The polymersomes were purified from the non-encapsulated enzyme by spin-diafiltration (Amicon Ultra Regenerated Cellulose centrifugal filters, MWCO = 100 000 g mol^{-1}). The volume was filled for 10 times with 0.1 M phosphate buffer pH 6.6 and centrifuged at 1500 g for 30 min at 0 °C (Thermo Scientific, Heraeus Megafuge 16R, TX-400 x 400 mL Swinging Bucket Rotor) until no enzymatic activity could be observed by the ABTS assay in the filtered volume. The final volume of the sample was 1.5 mL.

Preparation of empty polymersomes for cryo-TEM

To allow cryo-TEM imaging, a concentrated dispersion of polymersomes was prepared. To this end, 5 solutions consisting of 0.5 mg of ADEN and 0.5 mg of THYM were dissolved together in 0.5 mL of THF

in 5 round bottom flasks. The solvent was slowly evaporated on a rotary evaporator to generate thin films. Then, 1 mL of ultrapure water was introduced into a first round-bottom flask, which was placed in an ultrasound bath over a period of 6 h. The resulting dispersion was then transferred to the second round-bottom flask containing a thin film of both ADEN and THYM polymers and the self-assembly process was repeated. The process was repeated throughout the remaining 3 thin films yielding a dispersion of ADEN/THYM polymersomes of 2.5 mg ml^{-1} ADEN and 2.5 mg ml^{-1} THYM. Samples of the dispersions were imaged by cryo-TEM.

Mechanical stimulation of dispersions containing polymersomes for fluorescence spectroscopy, UV-Vis spectroscopy and luminescence experiments

To observe the response to shear, dispersions containing shear-responsive polymersomes were repeatedly aspirated and ejected through a 120 mm needle (Braun, Sterican 21Gx3⁄4”) by a hand-driven 5 mL syringe (BD Discardit II). The samples were repeatedly stimulated considering one stimulation cycle to be one aspiration of the dispersion and one ejection. When the response of the polymersomes was measured by fluorescence spectroscopy or UV-Vis spectroscopy, the cuvettes containing polymersome dispersions were placed in sample holders and were stimulated inside the instruments, covering the cuvettes with parafilm to avoid spilling. In the case of fluorescence spectroscopy measurements, 3 mL of the polymersome dispersions were passed through the needle and syringe and the mechanical stimulation was cycled throughout a period of 21 min with a frequency of 11.9 cycles per min. In the case of UV-Vis spectroscopy measurements and luminescence experiments, 1 mL of the polymersome dispersions were passed through the needle and syringe and the mechanical stimulation was cycled at 15.7 cycles per min. UV-Vis experiments were stimulated for 70 min and luminescence experiments for a maximum of 27 min.

Uptake of sodium fluorescein by ADEN/THYM polymersomes and release

0.1 mL of a dispersion of empty ADEN/THYM polymersomes was mixed with 1 mL of a 94.1 mg mL^{-1} solution of sodium fluorescein (0.25 M). The resulting dispersion was stimulated with a syringe needle by means of a hand driven syringe for a period of 51 min. Then the dispersion was dialysed for a period of 3 days (Spectra/Por 6 standard regenerated cellulose, pre-wetted dialysis tubing molecular weight cut-off = 1000 g mol^{-1}) against 1 L of ultrapure water. The water was exchanged each day once. The polymersomes were further purified from remaining free dye and micelles by size exclusion chromatography on Sepharose 2B using ultrapure water as the mobile phase. Then, the samples were stimulated and measured as explained in Supporting Information 8.

Determination of the influence of shear in microfluidics devices

Sodium fluorescein-filled polymersomes were flown through a self-prepared poly(dimethyl siloxane) microfluidics device with rectangular sections of 20 µm height and alternating widths of 100 and 20 µm (Supporting Information 5). The dispersions were introduced into the microfluidics device by a HSK soft Ject syringe of 3 mL through PE-5 tubing (Dimensions: 0.86 x 1.32 mm) (SCI Scientific commodities), and pumped by a KDS LEGATO 110 syringe pump at a flowrate was of 10 mL h^{-1}. Fluorescence intensity was measured with a Nikon Eclipse TS 100 inverted microscope.

Thermal release of sodium fluorescein from ADEN/THYM and HEX polymersomes

To estimate an approximate temperature for the melting of the nucleobase pairs, 3 mL of dispersions containing either sodium fluorescein-filled ADEN/THYM polymersomes or sodium fluorescein-filled HEX polymersomes were introduced into fluorescence spectroscopy cuvettes (disposable PMMA

cuvettes, d= 1 cm, Sigma-Aldrich). The cuvettes were sealed with parafilm and inserted into the Peltier-thermostated cuvette holder of the fluorescence spectrometer. The cuvettes were subjected to increasing temperatures of 20 °C, 30 °C, 40 °C, 50 °C, and 60 °C. The polymersomes were subjected to each temperature for a period of 10 minutes, and then the temperature was increased. Fluorescence emission spectra were recorded every 30 s during the whole experiment.

To observe the response of the polymersomes to thermal energy, 3 mL of dispersions containing either sodium fluorescein-filled ADEN/THYM or sodium fluorescein-filled HEX polymersomes were introduced into fluorescence spectroscopy cuvettes (disposable PMMA cuvettes, d= 1 cm, Sigma-Aldrich). The cuvettes were sealed with parafilm and inserted into the cuvette holder, which was thermostated at 50 °C. The release of sodium fluorescein was followed by fluorescence spectroscopy performing measurements every 30 s throughout a period of 20 minutes.

Colorimetric assays catalysed by HRP-containing nanoreactors

Pyrogallol assay: 50 µL of a nanoreactor dispersion, 0.475 ml of a hydrogen peroxide stock solution (212.7 nmol ml^{-1} in 0.1 M phosphate buffer (pH = 6.6)) and 0.475 mL of a pyrogallol stock solution (10 µmol ml^{-1} in 0.1 M phosphate buffer (pH = 6.6)) were introduced into a disposable semi-micro poly(methyl methacrylate) cuvette (path length: 1 cm). The cuvette was placed in a cuvette holder of an UV-Vis spectrometer at room temperature and an initial spectral scan from 300 to 600 nm was taken after 10 min of incubation. The sample was then sheared through a syringe needle by repeated cycles of aspiration and release in and out of a syringe, as described above. UV-Vis spectral scans from 300 to 600 nm were taken every 100 syringe pass cycles until a total of 1100. At a frequency of 15.7 cycles per minute, this corresponds to a total time of 70 min. In the case of the non-stimulated polymersomes, absorbance was recorded at 420 nm every 14 s for a period of 70 min.

ABTS assay: 50 µL of a nanoreactor dispersion, 0.475 mL of an ABTS solution (10 µmol ml^{-1} in 0.1 M phosphate buffer (pH = 6.6)) and 0.475 ml of a hydrogen peroxide stock solution (212.7 nmol ml^{-1} in 0.1 M phosphate buffer (pH = 6.6)) were introduced into a disposable semi-micro poly(methyl methacrylate) cuvette (path length: 1 cm). The cuvette was placed in a cuvette holder of an UV-Vis spectrometer at room temperature and an initial spectral scan from 300 to 600 nm was taken after 40 min of incubation. The sample was then sheared through a syringe needle by repeated cycles of aspiration and release in and out of a syringe, as described above. UV-Vis spectral scans from 300 to 600 nm were taken every 5 min through a total period of 30 min (at a frequency of 15.7 cycles per minute). In the case of the non-stimulated polymersomes, UV-Vis spectral scans from 300 to 600 nm were taken every minute through a total period of 30 min.

Chemoluminescence reactions catalysed by HRP-containing nanoreactors

Unless otherwise stated 25.0 mg (90.5 µmol) of ABEI were weighted together with 25.0 mg (114 µmol) of *p*-iodophenol. Thereafter, 2.5 mL of 0.1 M Tris-HCl buffer (pH 8.5) together with 0.75 mL of 0.4 M NaOH were added to obtain a stock solution that would be used for the luminescence reaction (final pH 9.74). 0.5 mL of this stock solution was added to a glass test tube. To this solution, 0.5 mL of a saturated solution of butanone peroxide (4.76 µmol mL^{-1} in 0.1 M Tris-HCl buffer (pH 8.5)) was added. Finally, 40 µL of a polymersome dispersion was added. The final concentration of the reagents was 13.4 µmol mL^{-1} ABEI, 16.8 µmol mL^{-1} p-iodophenol, and 2.28 µmol ml^{-1} butanone peroxide. The reactions were carried out at room temperature in a dark room. They were documented with a Nikon D7100 camera, equipped with a Nikon AF-S Micro NIKKOR 60mm 1:2:8 G ED lens. Photographs were

taken with dimensions of 4800 pixels width x 3200 pixels height, horizontal resolution of 300 dpi, vertical resolution of 300 dpi, F-stop f/4, exposure time of 8 s, ISO speed 1250, focal length of 60 mm, and maximum aperture of 3.2. Test tube and camera were placed in a distance of 25 cm by means of a clamp affixed to a scaffold and a tripod. Polymersomes were mechanically stimulated with a syringe and syringe needle as described above, and photographs were taken every 5 or 10 syringe passes. When the reaction mixture was not mechanically stimulated, photographs were recorded every 30 or 120 s. To compare the luminescence emission by ADEN/THYM nanoreactors and HEX nanoreactors, the concentration of HRP for every polymersome stock dispersion was measured by UV-Vis spectroscopy and the values were normalised accordingly in Fig. 4C and Supporting Information Fig. 14C. To quantify luminescence, each photograph was processed using ImageJ. The RGB images were converted into 32-bit images. Then, an area of the reaction mixtures was selected and the mean white pixel quantity per area unit was measured. To discriminate the influence of ambient light, neighbouring "dark" areas were measured and subtracted from the reaction mixture values. In the reactions where individual chemicals were exchanged, all other chemicals were employed in the concentrations described above. In experiments where ABEI was substituted by luminol or isoluminol, their concentration was of 20.9 µmol mL^{-1}. Butanone peroxide was substituted by hydrogen peroxide with a final concentration in the reaction test of 193.8 nmol ml^{-1}. In control experiments, non-encapsulated HRP was used as the catalyst. The reactions were carried out as described above as a means of comparison. Instead of the polymersome dispersions, 40 µL of 1.1 nmol mL^{-1} of free HRP in 0.1 M phosphate buffer pH 6.6 were introduced in the reaction mixtures. The results were normalised to the concentration of catalyst.

Gel formation catalysed by HRP-containing nanoreactors

Solutions of 43.11 mmol mL^{-1} hydrogen peroxide, 4.42 µmol mL^{-1} acrylamide, 0.117 µmol mL^{-1} *N*,*N*'-methylenebisacrylamide, 0.191 µmol mL^{-1} 2,4-pentanedione in 0.043 M phosphate buffer (pH 6.6) were prepared. Then, either 40 µL of HRP-loaded ADEN/THYM nanoreactors, or 100 µL of HRP-HEX nanoreactors were added. All dispersions were bubbled with argon for 10 min to remove oxygen. The nanoreactors were mechanically stimulated by ultrasound. Syringe aspiration does not work for this reaction, because it constantly introduces air into the reaction mixture. Oxygen inhibits radical polymerisation reactions. To stimulate the nanoreactors, they were ultrasonicated for 5 min in an ice-cold sonicator bath (Sonoswiss SW3) at an ultrasonic intensity of 4.1 W cm^{-2}. The progress of the reaction mixture was constantly assessed by turning the reaction flask and observing when the solution gelled. In the case of HEX nanoreactors, they were introduced in the sonicator bath for a period of 2 h. For comparison, reaction mixtures were also left at room temperature without ultrasonication for a period of 24 h.

Supporting Results and Discussion

Supporting Information 1. Synthesis and characterization of ADEN/THYM and HEX block copolymers

Amphiphilic block copolymers with tailored molecular weights for self-assembly into polymersomes were synthesised by reversible addition-fragmentation chain-transfer (RAFT) radical polymerisation. For this purpose, a poly(ethylene glycol) (PEG) macro-chain transfer agent (Supporting Information Scheme 1) was employed as the hydrophilic block to which hexyl methacrylate (HMA) and pentafluorophenyl methacrylate (PFPMA) were polymerised at a ratio 95:5 yielding activated ester block copolymers poly(ethylene glycol)-*b*-(poly(hexyl methacrylate)-*co*-poly(pentafluorophenyl methacrylate)). The block copolymers contained a randomly distributed activated ester in the hydrophobic block (Supporting Information Scheme 1).[5] Gel permeation chromatography measurements (GPC) showed polymers with relative narrow molecular weight distributions (dispersity *Đ*) (Supporting Information Fig. 5), as expected for a RAFT polymerisation.

With the purpose of synthesising polymers which would respond to mechanical stimulation by shear flow, and equivalent polymers which would remain inert, every activated ester block copolymer batch was split into three aliquots (Supporting Information Fig. 4A). One aliquot was functionalised with a primary amine modified adenine, the second aliquot with a thymine derivative featuring a primary amine and the third with hexylamine. This approach enabled the synthesis of polymers with equivalent content of complementary bases and control polymers with equivalent number of non-functional residues. ADEN, THYM, and HEX were obtained. The reactions were followed by ATR-FT-IR (Supporting Information Fig. 4B) and full functionalisation was confirmed by ^{19}F-NMR spectroscopy (Supporting Information Fig. 6). The polymers were characterised by GPC and ^{1}H-NMR spectroscopy (Supporting Information Fig. 5 and 7, and Supporting Information Table 1). Polymers with adequate block ratios for self-assembly into polymersome structures were obtained,[6] and the degree of functionalisation in the hydrophobic block was in good agreement with prediction. Although monomodal narrowly dispersed distributions were observed in the GPC traces for the activated ester containing block copolymers, ADEN and THYM showed shoulder formation at higher molecular weight. We hypothesise that this is due to disulphide bond formation between different polymer chains.[7]

Supporting Information Table 1 | Polymer library: Amphiphilic block copolymers containing PEG in the hydrophilic block and a random copolymer of poly(hexyl methacrylate) (PHMA) with poly(3-(adenine-9-yl)propyl methacrylamide) (PAPMAm) (ADEN), or poly(3-(thymine-1-yl)propyl methacrylamide) (PTPMAm) (THYM), or poly(hexyl methacrylamide) (PHMAm) (HEX).

	GPC				^{1}H NMR			
**Polymer**	M_n (g mol^{-1})	Đ	$f_{hydrophil}$ (% (w/w))	$f_{hydrophob}$ (% (w/w))	M_n (g mol^{-1})	$f_{hydrophil}$ (% (w/w))	$f_{hydrophob}$ (% (w/w))	Func. hydrophob. block (mol %)
**HEX**	$1·10^4$	1.30	21	79	$1.2·10^4$	14	86	4.6
**ADEN**	$1.4·10^4$	1.19	15	85	$8.4·10^3$	20	80	4.1
**THYM**	$8.7·10^3$	1.32	24	76	$7.5·10^3$	23	77	4.2

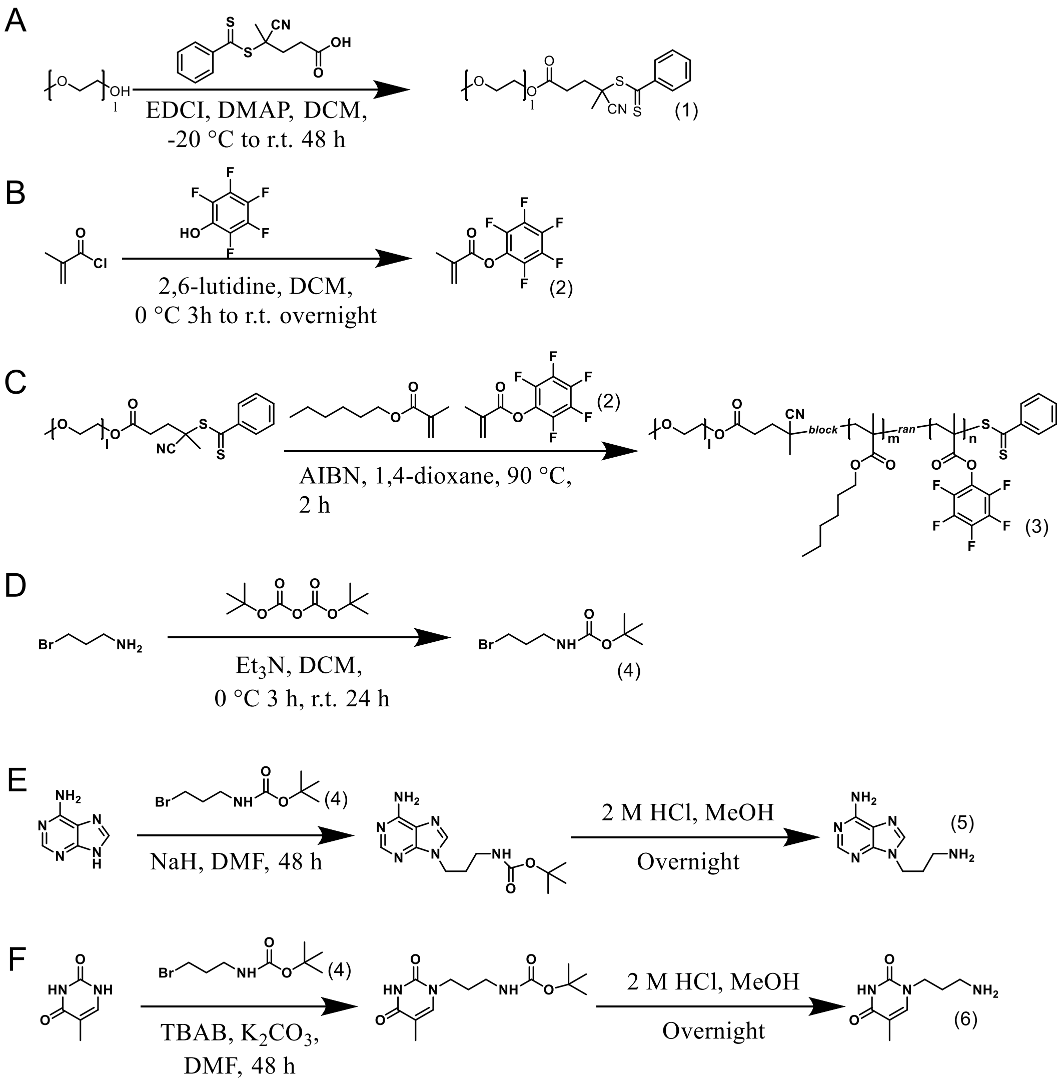

Supporting Information Scheme 1 | Synthesis of building blocks needed to prepare shear-responsive block copolymers. A. Synthesis of poly(ethylene glycol) methyl ether RAFT agent (**1**). **B**. Synthesis of pentafluorophenyl methacrylate (**2**). **C**. Synthesis of activated ester block copolymer of poly(ethylene glycol)-*b*-(poly(hexyl methacrylate)-*co*-poly(pentafluorophenyl methacrylate)) (**3**). **D**. Synthesis of *tert*-butoxycarbonyl (Boc)-protected 3-bromopropylamine (**4**). **E**. Synthesis of 3-(adenine-9-yl) propylamine (**5**). **F**. Synthesis of 3-(thymine-1-yl) propylamine (**6**).

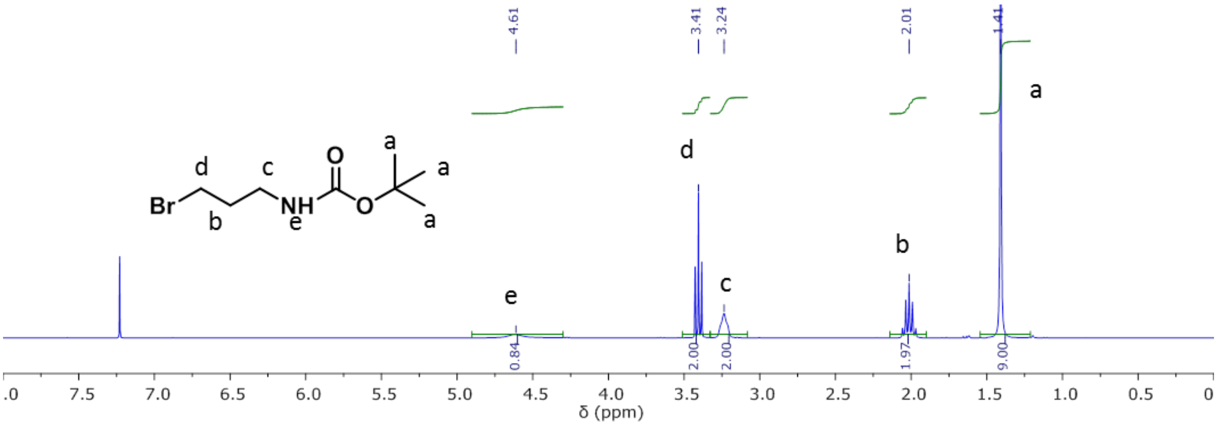

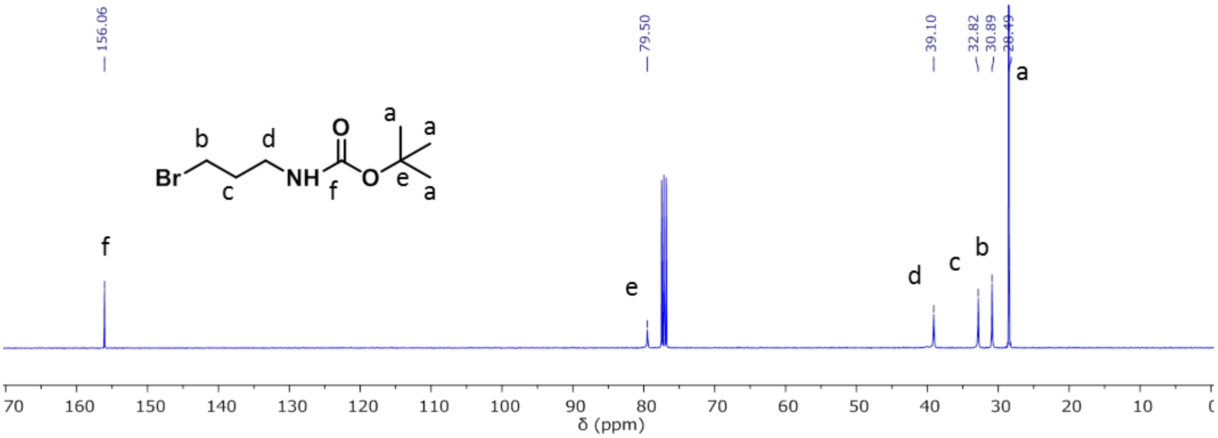

Supporting Information Figure 1 | NMR data of 3-(Boc-amino) propyl bromide in CDCl₃. A. ^{1}H NMR spectrum. **B.** ^{13}C NMR spectrum.

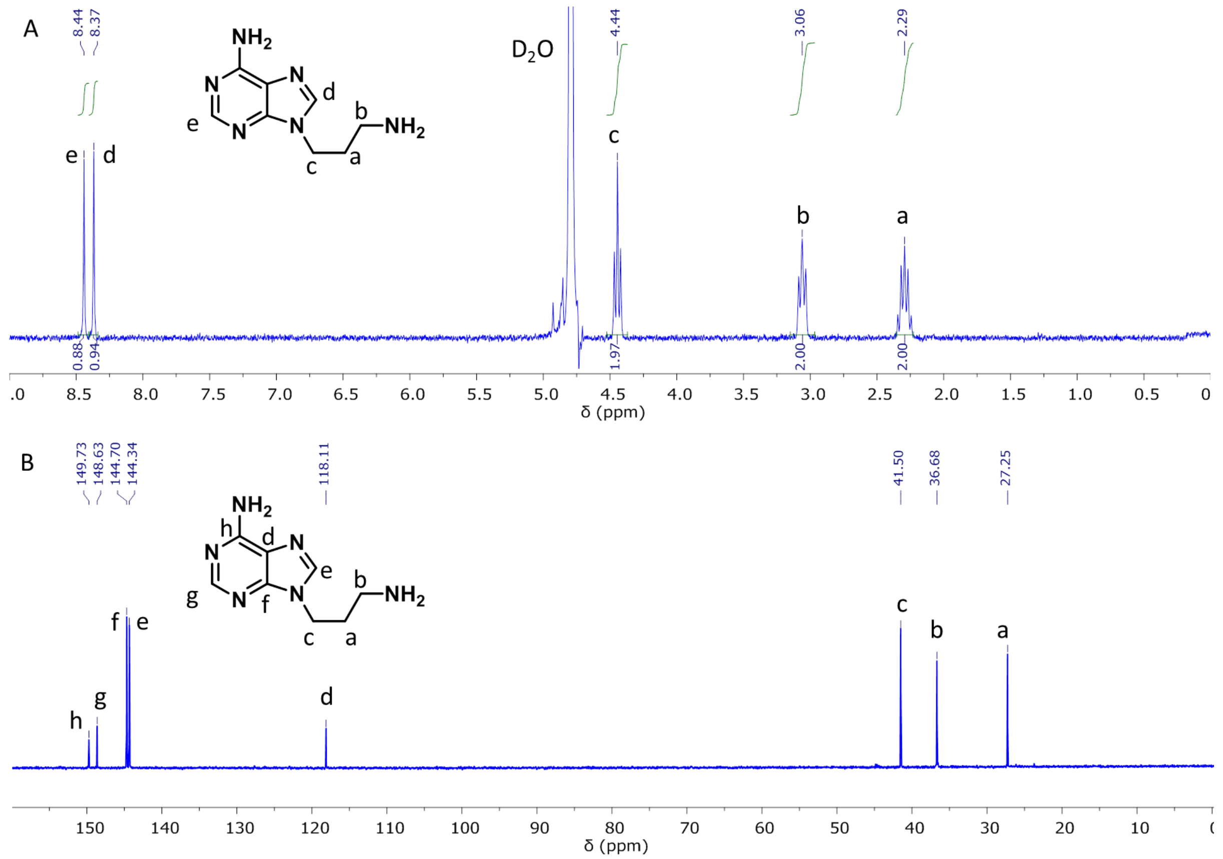

Supporting Information Figure 2 | NMR data of 3-(adenine-9-yl) propylamine in D_2O. A. ^{1}H NMR spectrum. **B.** ^{13}C NMR spectrum.

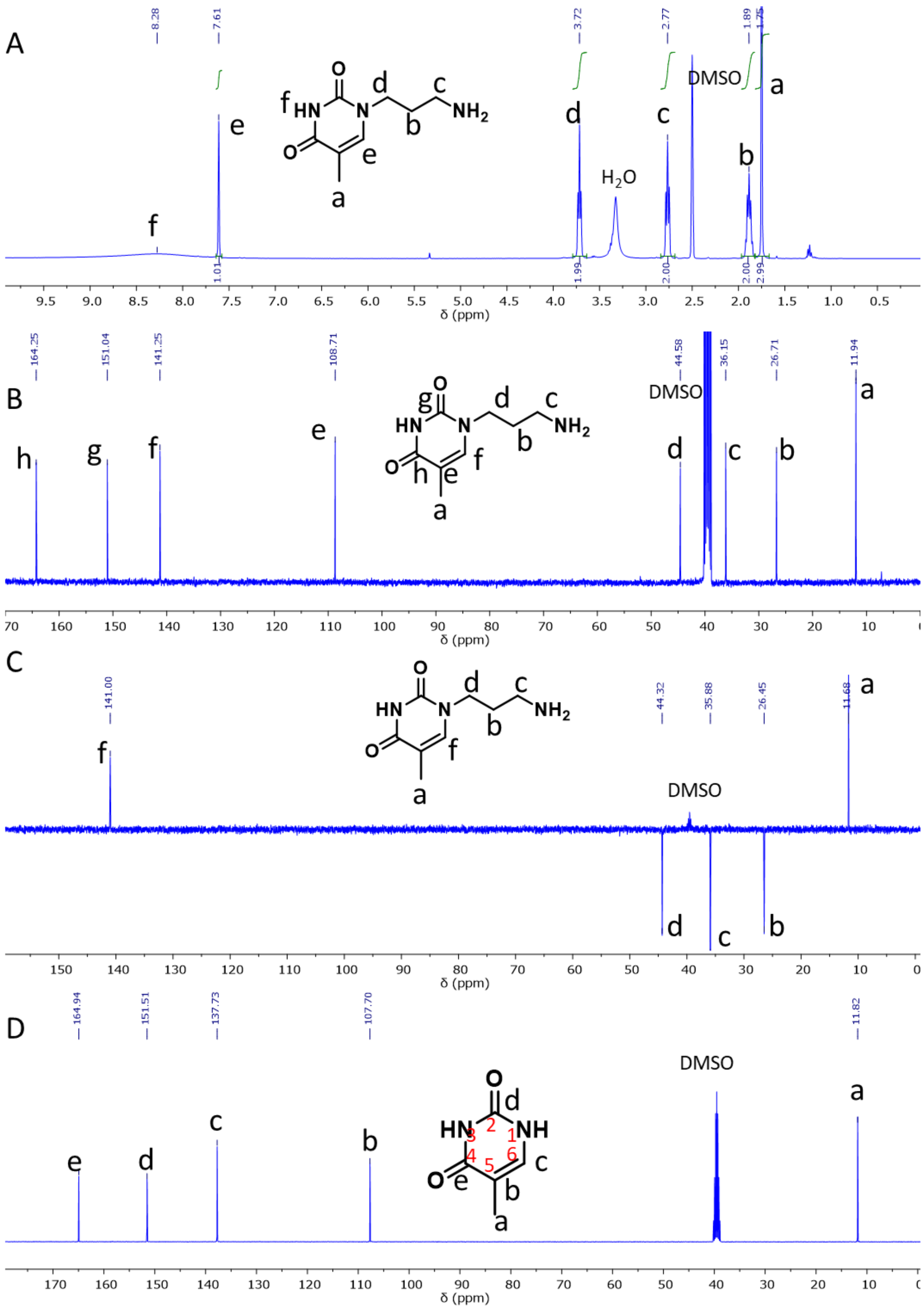

Supporting Information Figure 3 | NMR data of 3-(thymine-1-yl) propyl amine in d_6-DMSO. A. ^{1}H NMR spectrum. **B.** ^{13}C NMR spectrum. **C.** DEPT-135 spectrum. **D**. ^{13}C NMR spectrum of thymine. The latter allows concluding that thymine is functionalised only on the nitrogen in position 1, because a chemical shift of 3 ppm can be observed in position 6 of thymine upon its modification with propyl amine. In comparison, no considerable chemical shifts are observed in positions 2 and 4.

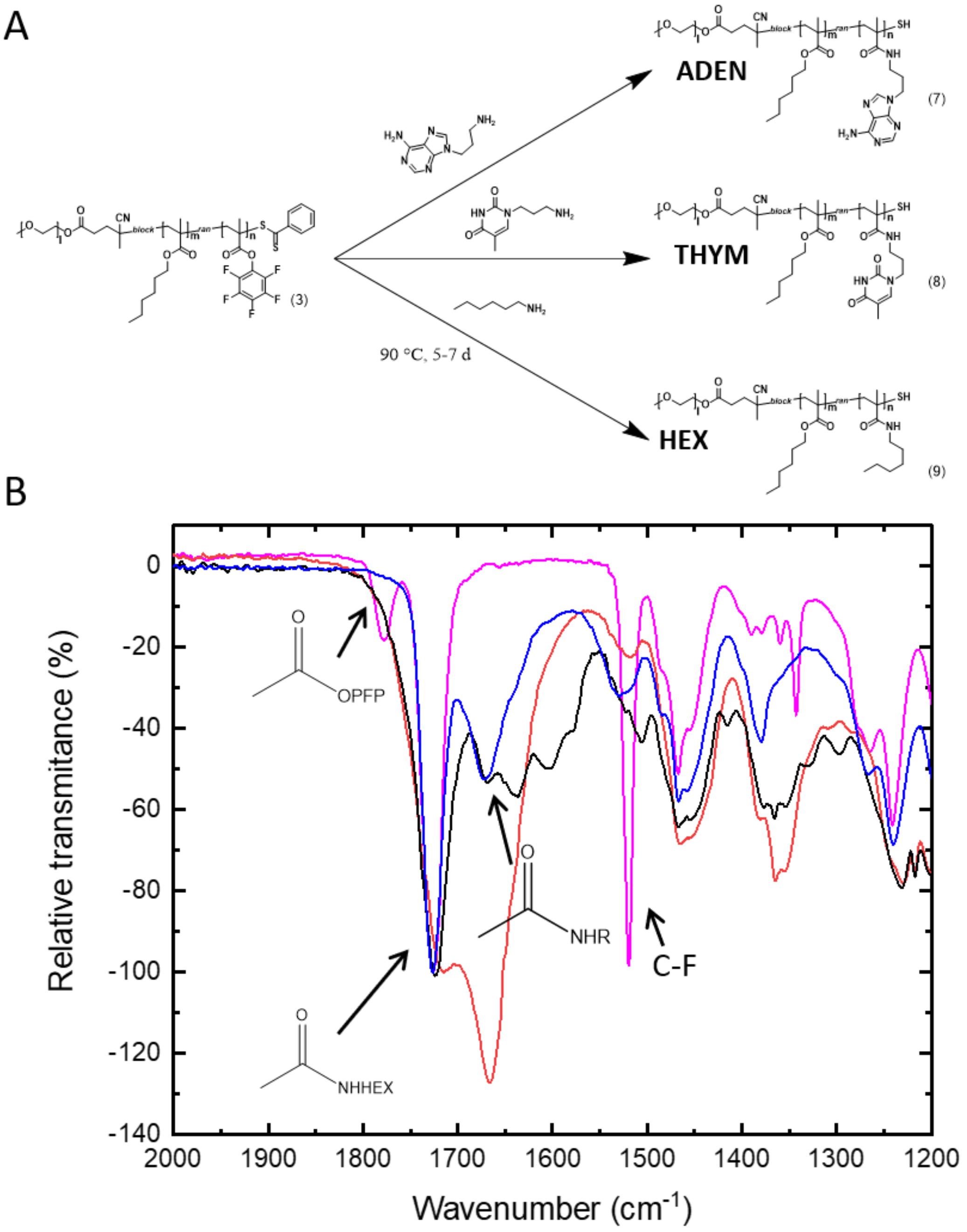

Supporting Information Figure 4 | Post-polymerisation functionalisation of amphiphilic block copolymers. A. Scheme of activated ester block copolymer activated ester functionalisation with primary amine modified adenine and thymine, as well as with hexylamine. **B**. ATR-FT-IR spectra of poly(ethylene glycol)-*b*-(poly(hexyl methacrylate)-*co*-poly(pentafluorophenyl methacrylate)) with a ratio of HMA:PFPMA 95:5 in the hydrophobic block before amidation (pink) and after amidation with the primary amine modified adenine (black), with the primary amine modified thymine (red) and with hexylamine (blue). Upon amidation, the absorption bands of the activated ester and the pentafluorophenyl C-F bonds disappear, while a signal that is typical for amide bonds appears. These changes allow concluding that the modification of the block copolymer with amines was successful.

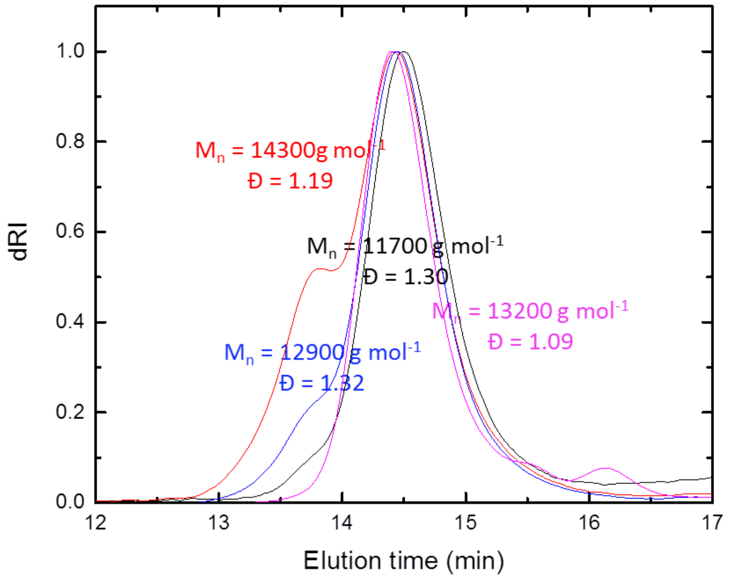

Supporting Information Figure 5 | GPC elugrams of functionalised block copolymers. GPC elugrams of ADEN (red), THYM (blue) and HEX (black) synthesised from one batch of activated ester block copolymer with a ratio HMA:PFMA 95:5. The GPC elugram of the precursor activated ester block copolymer is shown in pink. The pronounced shoulders at lower elution time (higher molecular weights) on the post-functionalised polymers could be due to dimerization of polymers via disulphide bond formation during the aminolysis.[7] The shoulder at longer elution time in the trace of the activated ester block copolymer comes from non-chain extended macro RAFT agent. It was removed in the next stages of the synthesis by the purification process.

Supporting Information 2. Determination of the degree of functionalisation by ^{19}F-NMR spectroscopy

For this purpose, 70 mg of every functionalised polymer were weighted and dissolved in a 10 mg mL^{-1} (54.32 µmol mL^{-1}) pentafluorophenol stock solution in $CDCl_3$ and subjected to ^{19}F-NMR spectroscopy. Only the peaks corresponding to pentafluorophenol were observed (Supporting Information Fig. 6) for all polymers, indicating that all PFPMA units had reacted with the primary amines.

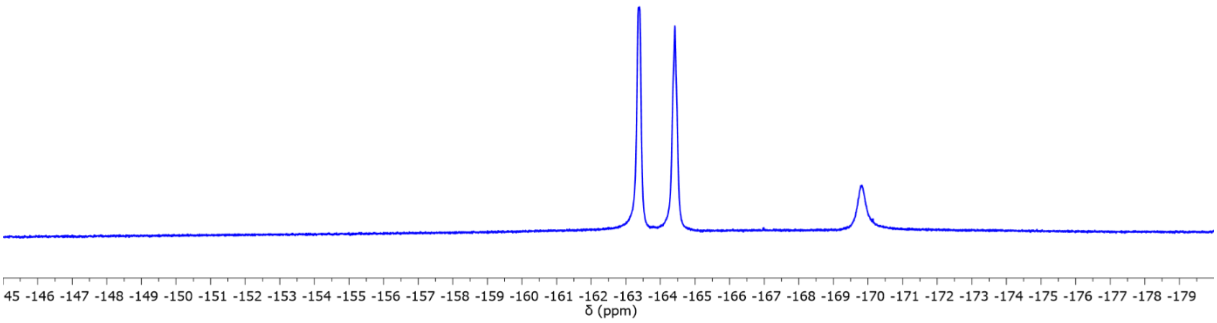

Supporting Information Figure 6 | ^{19}F-NMR spectrum of PEG-*b*-poly(HMA-*co*-adenine methacrylamide) (ratio of HMA:AM 95:5) in the presence pentafluorophenol as a standard. Only the peaks of the pentafluorophenol standard are present, indicating that the polymer did not contain any unreacted pentafluorophenyl ester groups. This allows concluding that full modification of the polymer with the nucleobase was achieved.

Supporting Information 3. Determination of number average molar mass, block ratio and degree of functionalisation by ^{1}H-NMR spectroscopy

The number average molar mass (M_n) of the PEG block was determined to be 1990 g mol^{-1} according to a previously described procedure.[8]

The number average molar mass of the functionalised block copolymers **7**, **8** and **9** was determined by Equation S1:

$$M_n = M_{n\,PEG} + \frac{3c}{2a} \cdot 170.25\ g\ mol^{-1} + \frac{3d}{2a} M_x \tag{S1}$$

With: *a* being the 3 methoxy end protons of the PEG block (3.36 ppm), *c* being the 2 methylen protons adjacent to the ester group of poly(hexyl methacrylate) (3.82 – 4.15 ppm), *d* being the 2 methylen protons adjacent to the amide groups of either poly(3-(adenine-9-yl)propyl methacrylamide), poly(3-(thymine-1-yl) methacrylamide), or poly(hexyl methacrylamide) (2.82 – 3.30 ppm), and M_x the molar mass of either TPMAm or APMAm.

The assignment of proton and the integrals can be found in Supporting Information Fig. 7.

The block ratio was described as the M_n percentage of the hydrophilic fraction ($\boldsymbol{f}_{\mathrm{hydrophil}}$) and the M_n percentage of the hydrophobic fraction ($\boldsymbol{f}_{\mathrm{hydrophob}}$) with Equation S2 and Equation S3:

$$f_{hydrophil}(\%) = \frac{M_{n\,PEG}}{M_n} \cdot 100 \tag{S2}$$

$$f_{hydrophob}(\%) = 100 - f_{hydrophil}(\%) \tag{S3}$$

The degree of functionalisation in the hydrophobic ($F_{\%}$) block could be determined by Equation S4:

$$F_{\%} = \frac{d}{c+d} \cdot 100 \tag{S4}$$

The results for the determination of number average molar mass (M_n), block ratio ($\boldsymbol{f}_{\text{hydrophil}}$, $\boldsymbol{f}_{\text{hydrophob}}$) and degree of functionalisation ($F_{\%}$) by ^{1}H-NMR spectroscopy are shown in Table 1.

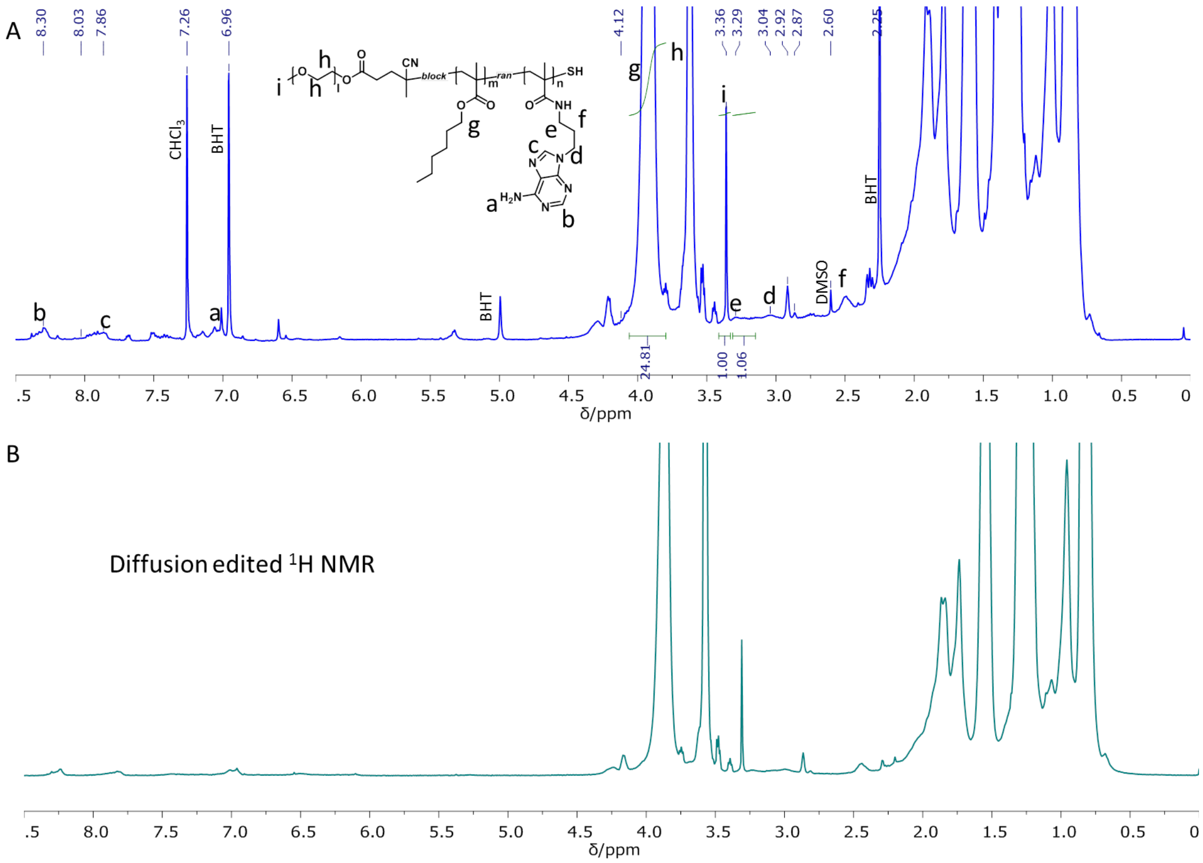

Supporting Information Figure 7 | ^{1}H NMR spectrum of ADEN/THYM to confirm the ratio of the two comonomers in the hydrophobic block and the M_n of the block copolymers. A. ^{1}H NMR spectrum. **B**. Diffusion edited ^{1}H NMR spectrum. Only resonances from polymer-bound species are observable and all signals of low molecular weight impurities are practically eliminated.

Supporting Information 4. Self-assembly and characterisation of self-assembled structures

Complementary ADEN and THYM were mixed at equimolar concentrations and self-assembled by the film rehydration method. To determine the hollow nature of the structures, the hydrodynamic radius was measured by dynamic light scattering (DLS), and the radius of gyration was measured by static light scattering (SLS). The results are summarised in Supporting Information Table 2, showing a ratio for these values (ρ= R_g/R_h= 0.96) close to 1.0 and above 0.77, accounting for hollow spheres, i.e. polymersomes.

Accurate membrane thickness (more precisely: the thickness of the hydrophobic leaflet of the membrane) was determined to be 15.5 ± 4.9 nm by measuring at 200 places on polymersomes imaged by cryo-TEM (2 measurements polymersome^{-1} on polymersomes which presented diameters above 300 nm, and 1 measurement polymersome^{-1} on the polymersomes below 300 nm) (Fig. 1).

The contour length (R_{cont}) of the polymer, i.e. the length of the polymer in a fully stretched conformation, was calculated as follows:

$$R_{cont} = D_p \cdot b \qquad \text{(S5)}$$

Where D_p is the degree of polymerization in the hydrophobic block, which was estimated to be of 35 units per polymer, and b is the segment length of a carbon-carbon bond in sp^3 hybridisation, 0.255 nm. R_{cont} was calculated to be of 8.937 nm. Given the value of the membrane thickness of 15.5 nm, we exclude that the polymers form an interdigitated monolayer membrane, and conclude that the polymers form a bilayer structure. This line of thought and the cryo-TEM images shown in Figure 1 also suggest that unlimalellar and not multilamellar vesicle walls were formed. The thickness of polymersome membranes composed of PEG-*b*-PHMA with similar M_n with non-complementary pendant groups has been reported previously with membrane thicknesses of aprox. 15 nm.[8]

Supporting Information Table 2 | Light scattering data of polymersomes

Polymersome composition	R_g (nm)	R_h (nm)	PDI (SD Mean^{-1})	$\rho = \frac{R_g}{R_h}$
**ADEN/THYM**	93	101	0.20	0.92
**HEX**	97	99	0.12	0.98

Supporting Information 5. Determination of shear stress in the microfluidic device

Device fabrication. We fabricated the microfluidic device from PDMS using soft lithography.[9] It consisted of one inlet that lead into a 100 µm wide channel which was confined to a length of 200 µm to form a 20 µm wide, 20 µm tall constriction before the channel widened again. This constriction was repeated three times. After the fluid passed all constrictions, it reached the outlet.

Observation with fluorescent microscope. The channels were observed through an inverted microscope Nikon Eclipse TS 100. The excitation wavelength for fluorescence was λ_{495nm} and the images were processed through a λ_{519nm} filter. The exposure time was set at 3s.

Calculation of shear stress in the constrictions as a function of flow rate

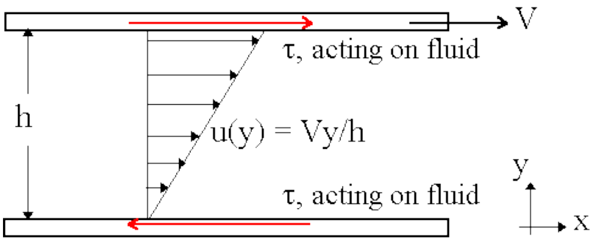

In a fluid circulating at a speed *v* in a direction *x*, the shear stress, τ, at a distance *y* from a plane surface is defined by Equation S6:

$$\tau(y) = \mu\left(\frac{\partial v(y)}{\partial y}\right) \quad \text{(S6)}$$

Where μ corresponds to the viscosity and *v* is the speed. We obtain the maximum shear stress that can be obtained from 4 walls at the centre of the channel (Equation S7).

$$\tau = 2\,\mu\left(\frac{v}{h/2}\right) + 2\,\mu\left(\frac{v}{w/2}\right) \quad \text{(S7)}$$

Where *v* is the speed in the centre of the channel, *h* is the height of the channel and *w* its width. Since the flowrate is defined by $Q = v \cdot h \cdot w$ we obtain Equation S8:

$$\tau = \frac{4\mu Q}{hw} \cdot \left(\frac{1}{h} + \frac{1}{w}\right) \quad \text{(S8)}$$

Hence, we obtain a correlation between the flowrate injected in the channel and the flowrate applied. In this case, for *h*=*w*=20 µm in water we obtain a conversion rate (Equation S9):

$$\frac{\tau}{Q} = 1.\cdot 10^{10}\ \frac{Pa}{m^3/s} \quad \text{(S9)}$$

Thus, having a flowrate *Q* of 10 mL h^{-1} ($2.78 \cdot 10^{-9}$ m^3 s^{-1}) implies a shear rate τ of 2.78 kPa; around 2 times higher than in the experiments performed with hand-driven syringes and needles. A more rigorous calculation, accounting for the fully developed flow profile in a channel with a square cross section, leads to almost identical results.

Supporting Information 6. Sodium fluorescein release studies

Sodium fluorescein is a fluorescent molecule in dilute state. When concentrated, it encounters itself in a self-quenching fluorescence regime. This property is commonly exploited to measure the release from the inner concentrated lumen of vesicles to the dilute bulk solution. To this end, 1 mL of a purified dispersion of either sodium fluorescein-filled HEX or ADEN/THYM polymersomes was introduced in a disposable semi-micro PMMA cuvette with a path length of 1 cm. Then, ultrapure water was added to obtain a final volume of 3 mL. The samples were mechanically stimulated at 20 °C, and fluorescence measurements were taken every 50 s by exciting the sample at $\lambda_{494\ nm}$ with an excitation slit width of 5 nm and an emission wavelength range of $\lambda_{450\ nm}$ to $\lambda_{650\ nm}$, with an emission slit of 5 nm and a scanning speed of 600 nm min^{-1}. If bubbles were observed in the cuvette, the sample was gently shaken to avoid interference with the fluorescence measurements. The quantification of the released sodium fluorescein from the lumen of the polymersomes to the bulk solution was realized as described in a previous report.[8]

Supporting Information 7. Intermittent shearing of fluorescein-loaded polymersomes

Sodium fluorescein-filled ADEN/THYM polymersomes were sheared through a syringe needle for 50 s, then left unperturbed for 4.6 min and sheared again for 7.6 min (Supporting Information Fig. 8). The fluorescence substantially increased when shear was initially applied, and the release of dye gradually became slower when the suspension rested. Even though polymersomes that had not been sheared at all are tight for fluorescein at room temperature, the resting polymersomes after the first round of shearing continuously released small amounts of fluorescein. When sheared for the second time the release rate increased and the polymersomes released the dye at a constant but slower rate than during the first mechanical stimulation. Therefore, the membranes could be switched by shear stress from a less permeable to a more permeable state for sodium fluorescein. These observations are in agreement with the nanoreactor experiments showed in the main manuscript.

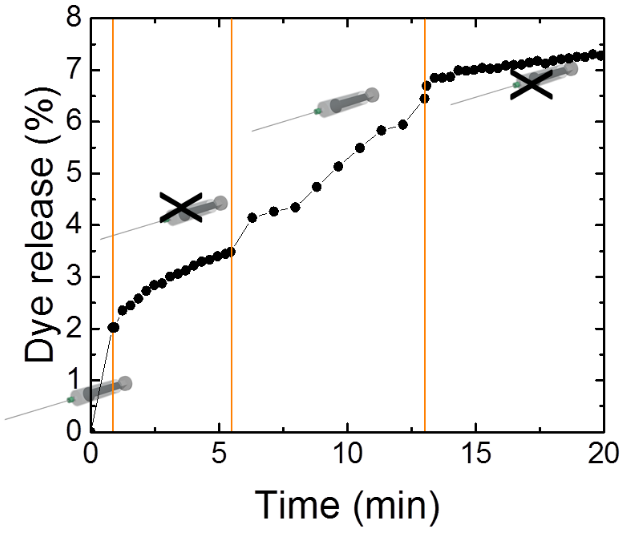

Supporting Information Figure 8 | Release of sodium fluorescein from ADEN/THYM polymersomes that were intermittently sheared through a syringe needle.

Supporting Information 8. Shear flow-induced uptake of sodium fluorescein into ADEN/THYM polymersomes and subsequent release of the dye upon shearing

To decipher if the release of dye was caused by an increase of membrane permeability or by shear flow-induced disassembly of polymersomes, a variety of experiments was performed. A dispersion of empty ADEN/THYM polymersomes was added to a concentrated sodium fluorescein solution and continuously sheared through a syringe needle for a period of 51 min. The dispersions were then dialysed until the washing water showed no fluorescence. The dispersion fluoresced, which shows that the polymersomes could uptake sodium fluorescein (Supporting Information Fig. 9A). Then, the system was sheared for a period of 21 min. The fluorescence increased linearly with time. Thus, the permeabilisation of the polymersome membrane was reversible (Supporting Information Fig. 9B). TEM micrographs after the uptake and release experiment show polymersomes, which suggests that the structure of the polymersomes remained intact during the mechanical stimulation (Supporting Information Fig. 9C). The nanoreactor experiments presented in the main manuscript also confirm that the polymersomes did not disassemble when they were sheared.

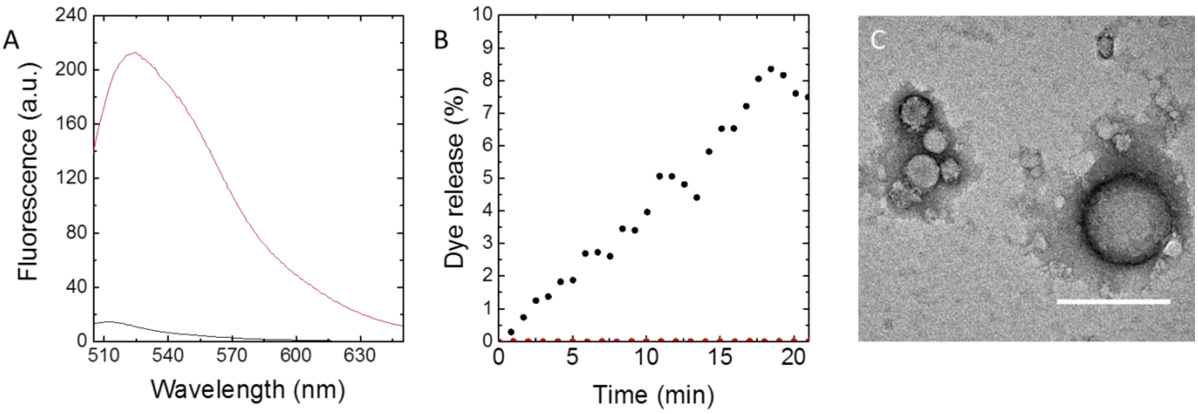

Supporting Information Figure 9 | Shear-induced uptake of sodium fluorescein into ADEN/THYM polymersomes and subsequent release of the dye upon shearing. A. Fluorescence emission spectra of empty polymersomes (black), and of polymersomes after continuously shearing a vesicle/sodium fluorescein mixture through a syringe needle for 50 min, followed by dialysis (red). The fluorescence indicates uptake of sodium fluorescein into the polymersomes. **B**. Release of sodium fluorescein from these polymersomes upon continuous shearing by means of a syringe (●), and in the absence of shearing (●). **C**. TEM micrograph of polymersomes after the uptake and release experiment. Scale bar: 200 nm.

Supporting Information 9. Determination of threshold activation shear flow of ADEN/THYM polymersomes

4 mL dispersions of sodium fluorescein-filled ADEN/THYM polymersomes were repeatedly aspirated and ejected through a 120 mm needle (Braun, Sterican 21Gx$^3/_4$”) by a syringe pump (Pump 11 Elite, Harvard Apparatus) driven 5 mL syringe (BD Discardit II). Each dispersion was aspirated and ejected 10 times at constant flowrates and was measured on a fluorimeter as explained in Supporting Information 6. To avoid mechanically induced annealing effects, the influence of each flowrate was measured with individual dispersions, meaning that the dispersions were not recycled for measuring other flowrates. Every experiment was carried out in triplicate and the mean values and standard deviations are reported in Supporting Information Fig. 10 together with the flowrates and the time of stimulation.

A

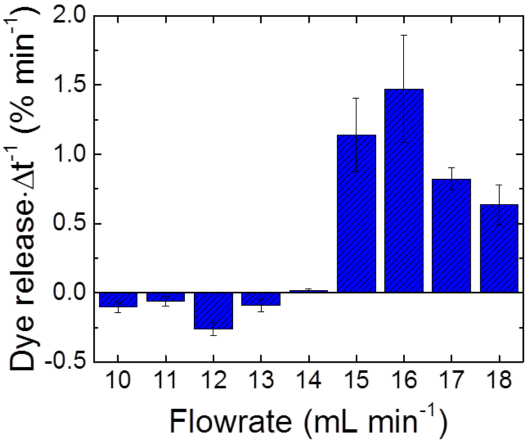

B

Flowrate (mL min^{-1})	Stimulation time (min)
10	8.0
11	7.3
12	6.7
13	6.2
14	5.7
15	5.3
16	5
17	4.7
18	4.4

Supporting Information Figure 10 | Measurement of the threshold flow needed to permeabilise shear-responsive polymersomes. A. Release of sodium fluorescein from ADEN/THYM polymersomes when continuously sheared through a syringe needle at defined flowrates over time. (mean value of n = 3 measurements; error = standard deviation). **B**. Table showing the time of shearing at the flowrates of panel A. As the total number of passages though the syringe needle was kept constant, the time of stimulation shortened with increasing flowrate.

Supporting Information 10. Estimation of the shear forces acting in the syringe and needle

The deformation of vesicles in hydrodynamic shear flow has been thoroughly studied experimentally and analysed mathematically for lipid vesicles, especially for giant liposomes, as they can be observed with a microscope. The deformation patterns and stress distributions are complex and often non-symmetrical.[10] They depend on several factors, incl. flowrates, the membrane viscosity, and the distance to the vessel walls. For polymers, the mobility of the amphiphiles in the membranes is more complicated than in lipid vesicles, as additional effects such as a perturbation of the polymer coil and chain entanglements come into play.[6a, 11] Therefore, and because the direct experimental observation of polymersome deformation in flow is difficult for vesicles on the nanoscale, we refrain from applying a detailed model from the liposome literature to compute the deformation and the associated stresses in the block copolymer membrane of the polymersomes. Instead, to estimate the shear forces that act on the polymersomes, we use a relatively simple geometric model for the deformation, which is the compression of the spherical vesicles into an oblate spheroid.

By considering the geometry of the syringe and syringe needle used to expose the polymersomes to shear stress, it is clear that the needle, i.e., the part of the experimental set-up with the smallest diameter, is the region where the strongest shear forces are generated. Therefore, this analysis was confined to the needle. For this purpose, the syringe needle was considered as a stainless-steel tube with a small diameter. The first step was to estimate the Reynolds number *Re* of the flow through the needle, which allowed to discriminate between laminar and turbulent flow:[12]

$$Re = \frac{\delta \cdot v_{av} \cdot D}{\eta} \tag{S10}$$

$$v_{av} = \frac{6Q}{\pi D^2} \tag{S11}$$

In Equation S10, δ is the density of the solution (water), *D* is the diameter of the syringe needle, η is the solution dynamic viscosity and v_{av} is the mean velocity of the fluid in the needle. In Equation S11 *Q* is the volumetric flowrate of the liquid going through the needle. Knowing the diameter of the needle (*D* = 0.65 mm) and the flowrates reported in Supporting Information Fig. 10, the corresponding Reynolds numbers are all smaller than 2600, which is the well-known threshold for the onset of turbulence in tubes,[13] the flow field in the needle is laminar. The analysis of the forces acting on a spherical particle in a parabolic flow is in general quite complex. To simplify the analysis, we assume that the polymersomes experience a simple shear flow. A simple shear flow is obtained in the case that the velocity gradient is constant. Instead, the velocity profile in the syringe needle is parabolic under laminar conditions:

$$v(r) = 2v_{av}\left(1 - \left(\frac{2r}{D}\right)^2\right) \tag{S12}$$

Where *r* is the radial coordinate from the centre of the needle. The modulus of the shear rate reads:

$$\left|\frac{dv}{dr}\right| = \frac{8v_{av}}{D}\left(\left(\frac{r}{D}\right)\right) \tag{S13}$$

The shear rate is a function of the radial coordinate, being maximum at the wall and equals to 0 at its centre. However, compared to the size of the polymersomes, it can be assumed that the change in shear rate with the radial coordinate is negligible, so it is reasonable to assume that the particles will experience simple shear conditions. Under simple shear conditions, it is possible to compute the traction that a particle with size *R* experiences. The surface traction f_t (which has the dimension of a pressure) that a spherical particle experiences is proportional to the velocity of the flow, according to the following equation[13]:

$$f_t = \frac{3}{R}\eta\left|\frac{dv}{dr}\right|r = \frac{24v_{av}}{R\cdot D}\left(\left(\frac{r}{D}\right)\right)r \tag{S14}$$

The maximum surface traction is achieved for r=D/4. The maximum values of the surface traction are plotted in Supporting Information Fig. 11 for various particle radii as a function of the flowrate in the needle. One can observe that the values for which a release are observed is inversely proportional to the size of the particles and are in the range of 20-60 kPa for particles with radii in the interval 100-300 nm. Furthermore, the compressional stresses acting on the particle are about half as large as the surface traction, a result that is rigorously true for extensional flow.[12] Given the quasi-spherical nature of the polymersomes, and the low stiffness of the hydrophobic leaflet mostly composed of PHMA, we hypothesise that the differential forces applied along their axis must induce their elongation. Thus, the elongation process may lead base pairs to unpair, increasing permeability towards small molecules. It should be furthermore noted that, as the fluid is injected in the needle, there is a sudden contraction when the fluid passes from the syringe to the needle. This leads to a *vena contracta*, i.e., a region with even higher flow velocity and shear stresses,[14] even though the polymersomes will be exposed to it for a very short time. Furthermore, when the same analysis is applied to the velocity profile inside the microfluidic channel, one can see that the maximum traction values exerted on polymersomes are about 2-3 times higher than those in the syringe.

In the case of the experiments carried out in the syringe at higher flowrate, 70 mL min^{-1}, the flow velocity in the needle is v = 7.41 m s^{-1}, calculated by dividing the volume of the fluid passing through the needle in a given time. This results in the Reynolds number equals $R_e \approx 3420$. Because this value is above 2600, which is the well-known threshold for the onset of turbulence in tubes,[13] the flow field in the needle was fully turbulent under these conditions. The average shear $<E>$ rate could be estimated from the following Equation S15:[15]

$$\langle E \rangle = \frac{4.2}{6\sqrt{16}}\sqrt{\frac{\delta \cdot \varepsilon}{\eta}} \tag{S15}$$

with ε being the energy dissipation rate, which was calculated by Equation S16:[13]

$$\varepsilon = 2f\frac{v^3}{D} \tag{S16}$$

where f is Fanning's friction factor, which could be calculated from the Equation S17 (by Churchill):[13]

$$\frac{1}{\sqrt{f}} = -4\log_{10}\left(0.27 \cdot \sigma + \left(\frac{7}{Re}\right)^{0.9}\right) \tag{S17}$$

In Equation S17, σ is the relative roughness of the material. Assuming the material of the needle being stainless steel, σ= 0.0023.

It is well known that, under turbulent conditions, it can be assumed that below Kolmogorov length scale, the motion of particles occurs under extensional flow, with a shear rate obtained from Equation S12.[16] This means that the particles were compressed along one equatorial plane and elongated along the perpendicular direction. The knowledge of the shear rate allows one to calculate the compressional and extensional stresses (f_c and f_e, respectively) acting on a particle. These are computed from Equations S18 and S19:[12]

$$f_c = \frac{3}{2}\langle E \rangle \eta \tag{S18}$$

$$f_e = \frac{3}{2}\langle E \rangle \eta \tag{S19}$$

By substituting the numbers obtained from previous calculations, the values obtained were f_c = 28.5 Pa and f_e = 57 Pa, respectively.

It should be kept in mind that the values obtained from Equations S18 and S19 are based on the mean shear rate. It is however very well known that turbulent motion is characterized by strong fluctuations in both velocity and energy dissipation.[15] Using a well-established multifractal model to estimate the probability density function of the energy dissipation rate,[17] one finds that there is a distribution of energy dissipations rates which fluctuate, and which can generate values a few orders of magnitude times higher than the mean value estimated with Equation S16. This means that compressive and extensional stresses substantially higher than those estimated using the average shear rate are present inside the syringe needle in turbulent flow.

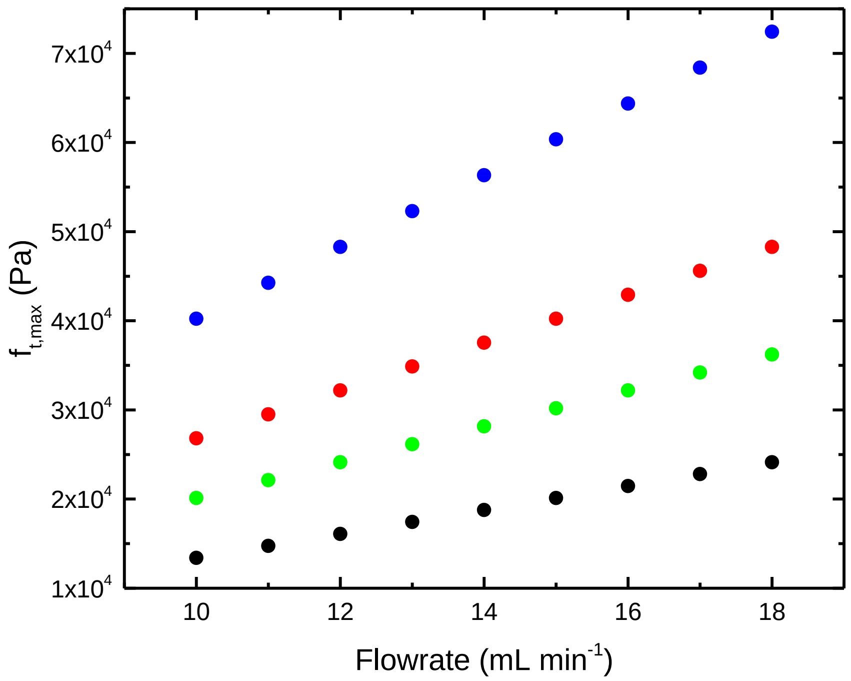

Supporting Information Figure 11 | Maximum values of the surface traction experienced by spherical particles in the syringe needle as a function of the flowrate. Values for hydrodynamic radii of 100 nm (blue), 150 nm (red), 200 nm (green), and 300 nm (black).

Supporting Information 11. Chemoluminescent reactions of HRP

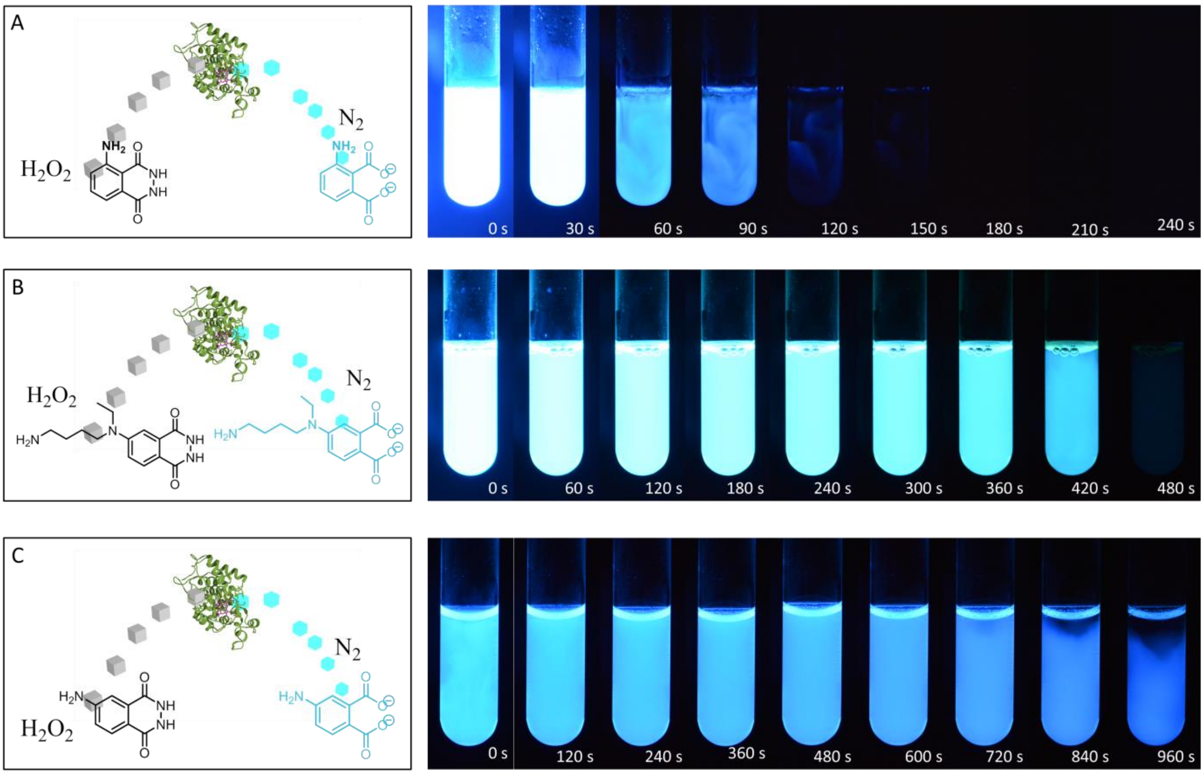

Supporting Information Figure 12 │Luminescent reaction of luminol with hydrogen peroxide catalysed by free HRP. A. Left: HRP-mediated reaction of luminol with hydrogen peroxide to produce a luminescent species. Right: Photographic sequence of a reaction in a test tube catalysed by non-encapsulated HRP. The reaction mixture starts to luminesce strongly within the first 30 s. Then, the luminescence decays as the reagents are consumed. **B**. Left: HRP-mediated reaction of ABEI with hydrogen peroxide to produce a luminescent species. Right: Photographic sequence of a reaction in a test tube catalysed by non-encapsulated HRP. **C**. Left: HRP-mediated reaction of isoluminol with hydrogen peroxide to produce a luminescent species. Right: Photographic sequence of a reaction in a test tube catalysed by non-encapsulated HRP.

Supporting Information 12. Selectivity of the polymersome membrane towards substrates of HRP

Alternatively to pyrogallol, we employed ABTS as a probe for the enzymatic activity of HRP-filled ADEN/THYM polymersomes. In the presence of hydrogen peroxide, HRP catalyses the formation of a green ABTS radical cation which absorbs in the visible spectrum.[18] The reaction mixture was sheared throughout a period of 30 min. The absorbance at 414 nm remained constant (Supporting Information Fig. 13). Thus, the nanoreactors were impermeable to ABTS impeding enzymatic activity. The systems that were not sheared also did not produce the product of the assay. Concluding, the membrane of ADEN/THYM polymersomes is selective to certain molecules. This can also be observed in the luminescence experiments. Apart from ABEI, other luminescent substrates were employed to assess if the membrane of the nanoreactors is substrate selective. HRP-filled ADEN/THYM polymersomes catalysed the reaction of luminol with 2-butanone peroxide in the presence of *p*-iodophenol right after mixing, *i.e.* before the nanoreactors were sheared (Supporting Information Fig. 14). These compounds

permeated through the membrane of the nanoreactors even in the absence of shear stress. The luminescence decayed within 6 min in the absence of shear stress. Interestingly, the reaction could be restarted when the system was sheared. Control reactions with HRP-filled HEX polymersomes showed very faint formation of luminescence in the absence of shear stress (Supporting Information Fig. 14C). When the system was sheared, luminescence was negligible. Most likely, this increase was due to a higher diffusion upon mixing and not due to a shear induced process. In contrast to ABEI, luminol can permeate through the ADEN/THYM polymersome membranes even in the absence of shear stress. However, the permeability of luminol increases when the reaction mixture is sheared.

The reaction of isoluminol with butanone peroxide in the presence of *p*-iodophenol did neither yield luminescence when a reaction mixture containing HRP-filled ADEN/THYM polymersomes was unperturbed, when it was sheared with a syringe nor when it was subjected to the high mechanical forces of an ultrasound bath (Supporting Information Fig. 15). In order to check that the reagents were intact, free HRP was added to the reaction mixture. Strong luminescence was observed. Thus, the reagents were intact, but isoluminol cannot cross the polymersome membrane. Moreover, the results confirm once more the integrity of the polymersome membrane during mechanical agitation, because ruptured polymersomes would release HRP and therefore trigger the chemoluminescent reaction.

To observe the influence of peroxides on the luminescent reactions, 2-butanone peroxide was substituted by hydrogen peroxide. Initial permeation in the absence of shear stress stimulation was observed for long periods in reactions with ABEI (5 min) and luminol (2 h). In both cases, the formation of luminescence increased with the application of shear to the system. Reactions of isoluminol together with hydrogen peroxide were similar than those conducted with 2-butanone peroxide and isoluminol.

To conclude, in contrast to ABEI and luminol, isoluminol does not permeate through the membrane of the shear-responsive nanoreactors, even when sheared. This observation is in line with literature reports showing that isoluminol does not cross through cell membranes while luminol is a membrane-permeable luminescent probe.[19] Though isoluminol and luminol are isomers, isoluminol presents higher polarity.[20] However, we observed that both luminol and ABEI permeate through the polymersome membranes.

We observed that the permeability of substrates increases in this order: ABTS < ABEI < luminol. In addition, we observed that hydrogen peroxide permeates more efficiently through the membranes than 2-butanone peroxide. Also, the molecular mass decreases in this order: ABTS > ABEI > luminol, and 2-butanone peroxide > hydrogen peroxide. Even though the polarity of small molecules seems to influence permeation, we hypothesise that steric hindrance is one of the main limitations to this process. Thus, the results strongly suggest that the permeability through ADEN/THYM polymersome membranes decreases with increasing radii of the compounds and that it can be increased by mechanical perturbation of the nucleobase-modified block copolymers. Therefore, the membranes show selectivity to certain compounds, which could be used to alter the substrate specificity of biotransformations.

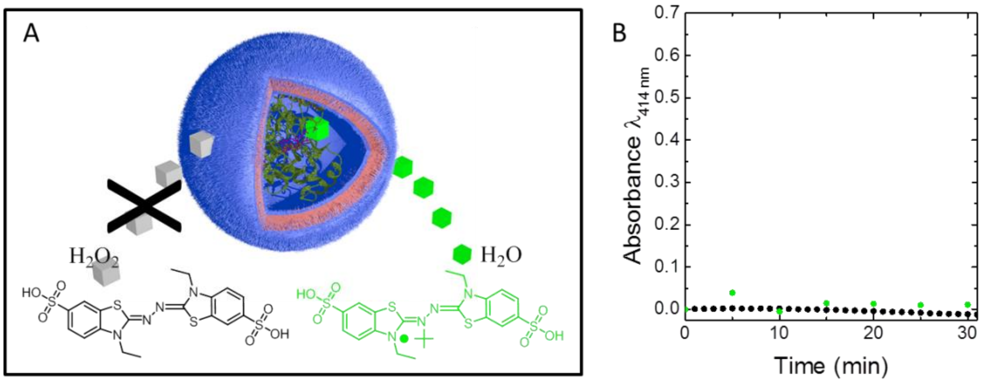

Supporting Information Figure 13 | ABTS assay catalysed by HRP-filled ADEN/THYM polymersomes. A. Reaction scheme. **B.** UV-Vis spectroscopic measurement of the formation of ABTS radical cation catalysed by continuously sheared HRP-filled ADEN/THYM polymersomes (green), and by non-sheared HRP-filled ADEN/THYM polymersomes (black).

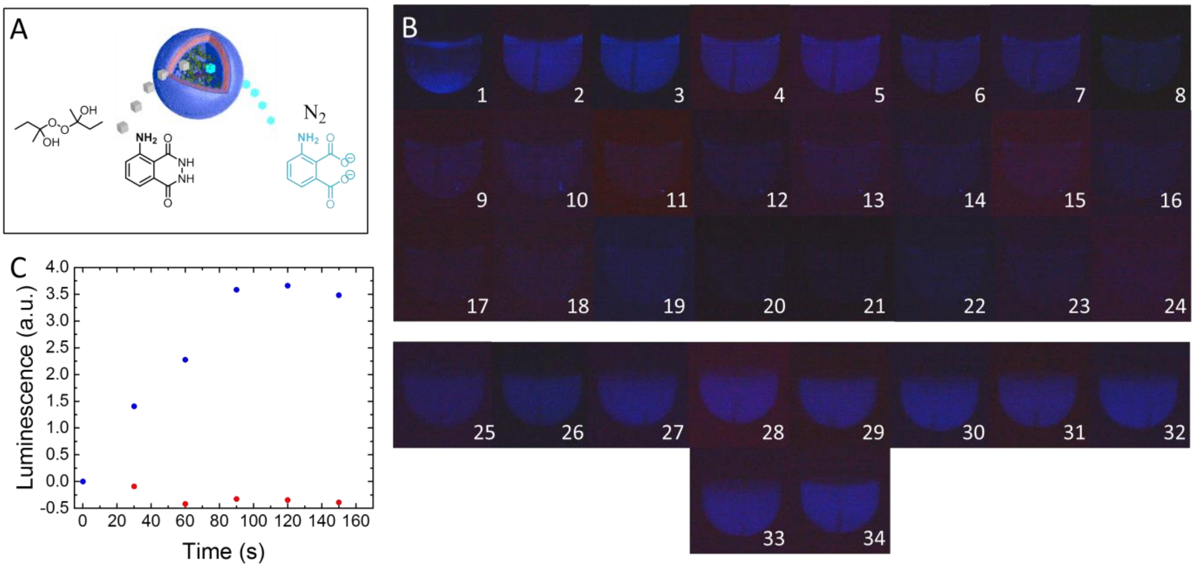

Supporting Information Figure 14 | Reaction of luminol with 2-butanone peroxide catalysed by HRP-filled ADEN/THYM polymersomes. A. Reaction scheme. **B**. Photo sequence of test tubes containing the reaction mixture including HRP-filled ADEN/THYM polymersomes. Photographs were taken every 30 s. 1-24: Reaction mixture after the initial mixing of reagents in the absence of shear. 25-34: Reaction mixture during continuous shearing. **C**. Formation of luminescence catalysed by continuously sheared HRP-filled ADEN/THYM polymersomes (blue) and by continuously sheared HRP-filled HEX polymersomes (red). The brightness of the photos was increased by 60 % for illustration purposes.

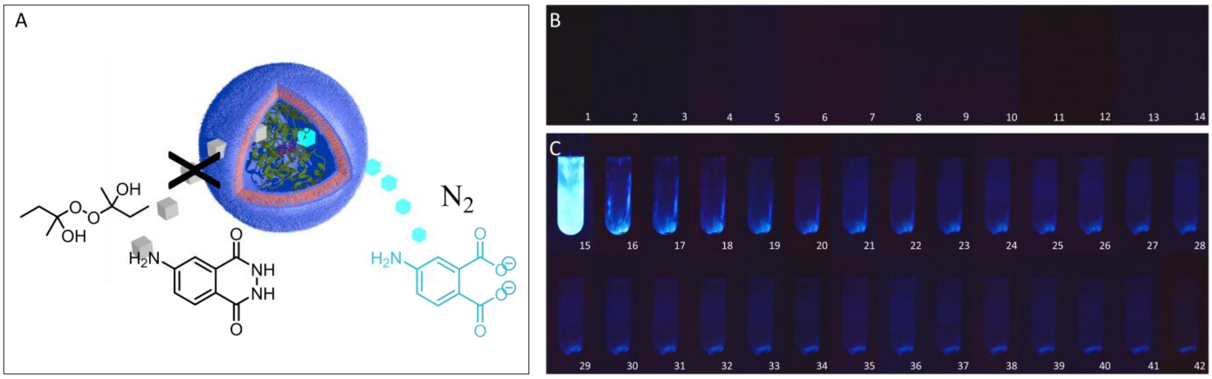

Supporting Information Figure 15 | Reaction of isoluminol with 2-butanone peroxide catalysed by HRP-filled ADEN/THYM polymersomes. A. Reaction scheme. **B**. Photo sequence of a test tube containing the reaction mixture including HRP-filled ADEN/THYM polymersomes. 1-11: The reaction mixture was continuously sheared through a syringe needle, and photographs were taken every 60 s. 12-14: The reaction mixture was sonicated in an ultrasound bath, and photographs were taken every 60 s. The reaction did not luminesce, indicating that the polymersome membrane did not become permeable for isoluminol. **C**. As a control to show that the reagents are intact, free HRP was introduced to the reaction mixture of the above experiment, and photographs were taken every 30 s. Luminescence formation was catalysed until the reagents were consumed. The brightness of the photos was increased by 50 % for illustration purposes.

Explanation of Supporting Information Video

The video shows the formation of an acrylamide gel by HRP-filled ADEN/THYM polymersomes upon shearing by means of an ultrasound bath as explained in the methods section of the main manuscript. Two reaction mixtures are shown, one that is introduced in an ice-cold sonicator bath (left) and a second reaction mixture that is left unperturbed (right). The dispersion of the left formed a gel after sonication, while the unperturbed sample remained in liquid. The video was accelerated 64x during the ultrasonication.

Supporting References

[1] a) S. Balog, *WO2020011949A1* **2020**; b) S. Balog, L. Rodriguez-Lorenzo, C. A. Monnier, B. Michen, M. Obiols-Rabasa, L. Casal-Dujat, B. Rothen-Rutishauser, A. Petri-Fink, P. Schurtenberger, *J. Phys. Chem. C* **2014**, *118*, 17968-17974.

[2] O. Rifaie-Graham, S. Ulrich, N. F. B. Galensowske, S. Balog, M. Chami, D. Rentsch, J. R. Hemmer, J. Read de Alaniz, L. F. Boesel, N. Bruns, *J. Am. Chem. Soc.* **2018**, *140*, 8027-8036.

[3] A. J. Brouwer, S. J. Mulders, R. M. Liskamp, *Eur. J. Org. Chem.* **2001**, *2001*, 1903-1915.

[4] H. J. Spijker, A. T. J. Dirks, J. C. van Hest, *Polymer* **2005**, *46*, 8528-8535.

[5] aM. Eberhardt, R. Mruk, R. Zentel, P. Théato, *Eur. Polym. J.* **2005**, *41*, 1569-1575; bA. Das, P. Théato, *Chem. Rev.* **2015**, *116*, 1434-1495; cM. Scherer, C. Kappel, N. Mohr, K. Fischer, P. Heller, R. Forst, F. Depoix, M. Bros, R. Zentel, *Biomacromolecules* **2016**, *17*, 3305-3317.

[6] aF. Itel, M. Chami, A. Najer, S. Lörcher, D. Wu, I. A. Dinu, W. Meier, *Macromolecules* **2014**, *47*, 7588-7596; bD. E. Discher, A. Eisenberg, *Science* **2002**, *297*, 967-973.

[7] J. Xu, J. He, D. Fan, X. Wang, Y. Yang, *Macromolecules* **2006**, *39*, 8616-8624.

[8] O. Rifaie-Graham, S. Ulrich, N. F. Galensowske, S. Balog, M. Chami, D. Rentsch, J. R. Hemmer, J. Read de Alaniz, L. F. Boesel, N. Bruns, *J. Amer. Chem. Soc.* **2018**, *140*, 8027−8036.

[9] aY. Xia, G. M. Whitesides, *Annu. Rev. Mater. Sci.* **1998**, *28*, 153-184; bJ. C. McDonald, D. C. Duffy, J. R. Anderson, D. T. Chiu, H. Wu, O. J. Schueller, G. M. Whitesides, *Electrophoresis* **2000**, *21*, 27-40.
[10] D. Abreu, M. Levant, V. Steinberg, U. Seifert, *Adv. Colloid Interface Sci.* **2014**, *208*, 129-141.
[11] H. Bermudez, A. K. Brannan, D. A. Hammer, F. S. Bates, D. E. Discher, *Macromolecules* **2002**, *35*, 8203-8208.
[12] C. Pozrikidis, *Introduction to theoretical and computational fluid dynamics*, Oxford university press, **2011**.
[13] D. W. Green, R. H. Perry, *Perry's chemical engineering handbook*, 8th ed., McGraw-Hill Professional, New York, NY, USA, **2007**.
[14] B. A. Aguado, W. Mulyasasmita, J. Su, K. J. Lampe, S. C. Heilshorn, *Tissue Eng., Part A* **2012**, *18*, 806-815.
[15] G. K. Batchelor, *The theory of homogeneous turbulence*, Cambridge university press, **1953**.
[16] G. K. Batchelor, *J. Fluid Mech.* **1980**, *98*, 609-623.
[17] M. U. Bäbler, M. Morbidelli, J. Bałdyga, *J. Fluid Mech.* **2008**, *612*, 261-289.
[18] P. C. Verlander, in *Nonisotopic DNA Probe Techiques* (Ed.: L. J. Kricka), Academic Press, Inc., San Diego, CA, USA, **1992**, pp. 185-201.
[19] aD. R. Mumbengegwi, Q. Li, C. Li, C. E. Bear, J. F. Engelhardt, *Mol. Cell. Biol.* **2008**, *28*, 3700-3712; bH. Lundqvist, C. Dahlgren, *Free Radical Biol. Med.* **1996**, *20*, 785-792.
[20] V. Jancinová, K. Drábiková, R. Nosál, L. Racková, M. Májeková, D. Holománová, *Redox Rep.* **2006**, *11*, 110-116.
